# Tuning the free energy of host–guest encapsulation by cosolvent†

**DOI:** 10.1039/d5cp00661a

**Published:** 2025-04-22

**Authors:** Melinda Nolten, Kay T. Xia, Simone Pezzotti, Gerhard Schwaab, Robert G. Bergman, Kenneth N. Raymond, F. Dean Toste, Teresa Head-Gordon, Wan-Lu Li, Martina Havenith

**Affiliations:** a Department of Physical Chemistry II, Ruhr University Bochum 44801 Bochum Germany martina.havenith@rub.de; b Chemical Sciences Division, Lawrence Berkeley National Laboratory Berkeley CA 94720 USA rbergman@berkeley.edu raymond@socrates.berkeley.edu fdtoste@berkeley.edu thg@berkeley.edu; c Department of Chemistry, University of California Berkeley CA 94720 USA; d Kenneth S. Pitzer Theory Center and Departments of Bioengineering and Chemical and Biomolecular Engineering, University of California Berkeley CA 94720 USA; e Department of Chemical and Nano Engineering, University of California San Diego CA 92093 USA wal019@ucsd.edu

## Abstract

Supramolecular hosts create unique microenvironments which enable the tuning of reactions *via* steric confinement and electrostatics. It has been shown that “solvent shaping inside hydrophobic cavities” is an important thermodynamic driving force for guest encapsulation in the nanocage host. Here, we show that even small (5%) changes in the solvent composition can have a profound impact on the free energy of encapsulation. In a combined THz, NMR and *ab initio* MD study, we reveal that the preferential residing of a single DMSO molecule in the cavity upon addition of ≥5% DMSO results in a considerable change of Δ*S* from 63–76 cal mol^−1^ K^−1^ to 23–24 cal mol^−1^ K^−1^. This can be rationalized by reduction of the cavity volume due to the DMSO molecule which resides preferentially in the cavity. These results provide novel insights into the guest–binding interactions, emphasizing that the entropic driving force is notably influenced by even small changes in the solvent composition, irrespective of changes in metal ligand vertices. Having demonstrated that the local solvent composition within the cage is essential for regulating catalytic efficiency, solvent tuning might enable novel applications in supramolecular chemistry in catalysis and chemical separation.

## Abstract 2

Experimental and theoretical study of [Ga_4_L_6_]^12−^ and [In_4_L_6_]^12−^ investigating guest encapsulation. Our study reveals that solvent composition, crucially affects entropy and guest binding, opening the door for tuning host–guest interactions.

## Graphical abstract

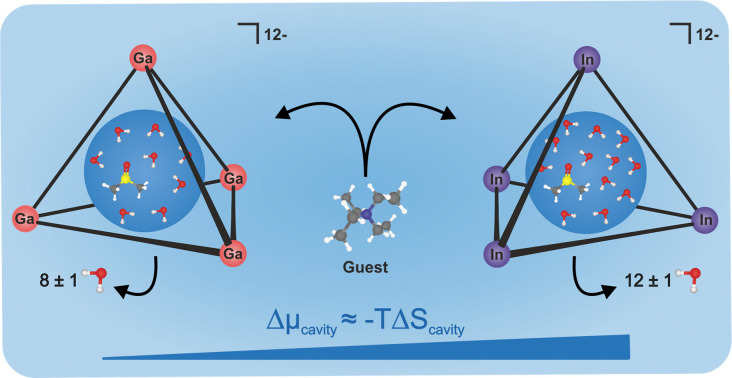

## Introduction

Tuning reactions in nanocages is of major interest for applications in chemical and materials science.^1–3^ Studies of host–guest dynamics have demonstrated high binding affinities and selectivity, and hosts have been shown to perform catalysis,^4–9^ chemical separations^10–12^ and cargo-transport.^13–15^ The organization of small molecules within confined cavities is different from that of species in bulk and is also of interest in studies of enzyme active sites, graphitic and zeolite pores, nanochannels, and the various applications of these systems.^16–22^ Understanding water organization within these cavities will aid the design of systems for purification, desalination, and the development of hydrophobic materials, along with other related technologies.^23–26^ The study of solvent organization in confined spaces has been conducted for a variety of enzymes and supramolecular materials through the use of guest–binding experiments.^27–32^ Modular supramolecular host assemblies provide an advantageous tool for these studies as they are structurally simpler than enzymes and can be synthetically tuned, enable greater control over their properties and the use of a wide range of spectroscopic and computational tools.

**Fig. 1:**
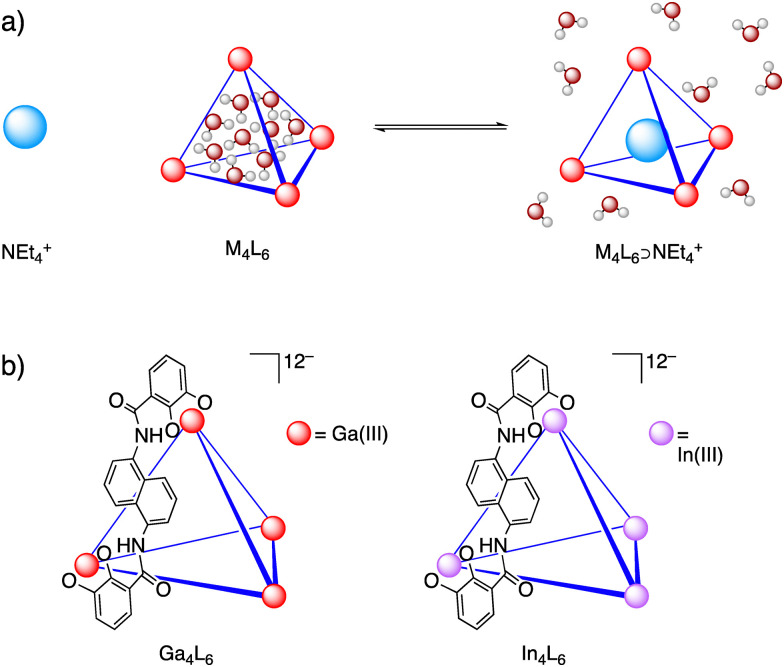
Tetrahedral M_4_L_6_ supramolecular host assemblies. (a) Scheme illustrating water organization inside the host cavity and proposed structural basis for entropic increase upon displacement of encapsulated water by a cationic guest molecule. (b) Structures of [Ga_4_L_6_]^12−^ and [In_4_L_6_]^12−^. Counterions are K^+^.

The water-soluble, highly anionic tetrahedral host assembly [Ga_4_L_6_]^12−^ has achieved catalytic rate accelerations rivaling those of enzymes, with entropically favorable guest encapsulation promoted by solvent shaping.^33–40^ The noncovalent host–guest interactions of the [Ga_4_L_6_]^12−^ system and their implications on catalysis and organization of substrates and solvents have been studied in a joint effort *via* experiment and theory.^9,33,41–47^ To further investigate the dynamics of encapsulated water, the spectra of these water molecules within the host cavity can be elucidated by introducing a strongly binding cationic tetraethylammonium guest (NEt_4_^+^). NEt_4_^+^ exhibits a significantly stronger affinity for residing in the interior of the host cavity compared to the exterior, effectively displacing water from within the host upon encapsulation.^46–50^ The differences in THz spectra of aqueous [M_4_L_6_] in the presence and absence of NEt_4_^+^ enable observation of the encapsulated water molecules, granting access to the unique properties of hydration water within the cavity environment. [Ga_4_L_6_]^12−^ has previously been shown to encapsulate 9 ± 1 water molecules within its cavity, which are structurally and dynamically distinct from any known phase of water.^51^ The release of these unusual encapsulated molecules creates a strong thermodynamic driving force for the high-affinity binding of guests in aqueous solution (Fig. 1a).^51^

The [Ga_4_L_6_]^12−^ host has been synthetically diversified into a series of structurally similar M_4_L_6_ hosts, and structure–activity relationships studies have been conducted to inform the role of each of the components of the assembly.^48,49,52,53^ Among these, the [In_4_L_6_]^12−^ host provides an isostructural comparison to [Ga_4_L_6_]^12−^, facilitating the investigation of metal vertex effects on solvent organization, guest binding, and catalytic reactivity (Fig. 1b). While naphthalene walls formed by the host's ligand are likely the primary contributor to the hydrophobicity of the cavity, the effect of the metal vertices on the cavity environment and solvation of the host is unclear. Despite being isostructural and having the same overall charge, [Ga_4_L_6_]^12−^ and [In_4_L_6_]^12−^ have significantly different solubilities: 100(1) mM and 4.2(3) mM in water respectively, while both are highly soluble in DMSO. This difference implies a considerable change in solvation induced by the difference in metal vertices as shown previously.^43,49^

While the choice of metal in the host system is suspected to significantly influence thermodynamics and encapsulation rates, it is not the sole determining factor: for the total free energy, a delicate balance exists among host, guest, and solvent interactions, which all have an impact on the encapsulation and the catalytic rate. Previous studies on the microsolvation of various guests by distinct solvent molecules within the cavities of both [Ga_4_L_6_]^12−^ and [In_4_L_6_]^12−^ investigated the relationship between solvation dynamics and reaction rates.^9,33,43,44^ These studies revealed that a close fit of the guest within the cavity, achieved by displacing surrounding solvent molecules, lowers the energy barrier for encapsulation. Consequently, any changes in microsolvation directly impacts catalytic rates, as the number of solvent molecules within the cavity adapts.^44^ However, solvent molecules contribute more than just structural occupancy; coordinated water may also act as a catalytic agent, highlighting the active roles of solvent molecules within the cavity.^54^

While these studies were focusing exclusively on single component solvents such as water or methanol, we show that even small partial contributions of additional solvents in mixtures of water and organic solvents can have a major impact on encapsulation. So far encapsulation efficiency has been attributed primarily to metal–ligand design, our experiments in DMSO/water mixture demonstrate that not only the presence of solvent but also its composition within the host cavity is crucial. We find that even a few co-solvent molecules can significantly alter the free energy of encapsulation, allowing precise tuning of the thermodynamic driving force by optimizing the mixture composition. Hence solvent tuning may provide a new approach for supramolecular hosts that are poorly or non-soluble in water and to enhance thermodynamic driving forces for encapsulation.

## Results

### Solvent structure within host cavities

THz spectroscopy was applied to the host–guest system to elucidate the water solvation of the host cavity and the effect of the metal vertices. Unless stated otherwise, all spectra were measured in a frequency range between 30 cm^−1^ and 430 cm^−1^ with a nominal spacer thickness of ∼25 μm. Due to the low solubility of [In_4_L_6_]^12−^ in pure water but high solubility in DMSO, we used a mixture of DMSO and water (1 : 9, V/V) for measurements, yielding a molar concentration of *c* ≈ 10 mM. For comparison, [Ga_4_L_6_]^12−^ was measured with the same concentration and the same composition of DMSO in solution as for [In_4_L_6_]^12−^, and these results were compared to reproduced measurements of [Ga_4_L_6_]^12−^ in ultrapure water as reported in ref. 51.

The absorption coefficients *α*_sample_ were first determined for each sample, as described in the ESI† (eqn (S7) and (S9)). Subsequently, the obtained spectra of *α*_sample_ (Fig. S18–S20, ESI†) were corrected by a scaled water vapor spectrum to minimize the absorption of residual air in the optical path.

**Fig. 2:**
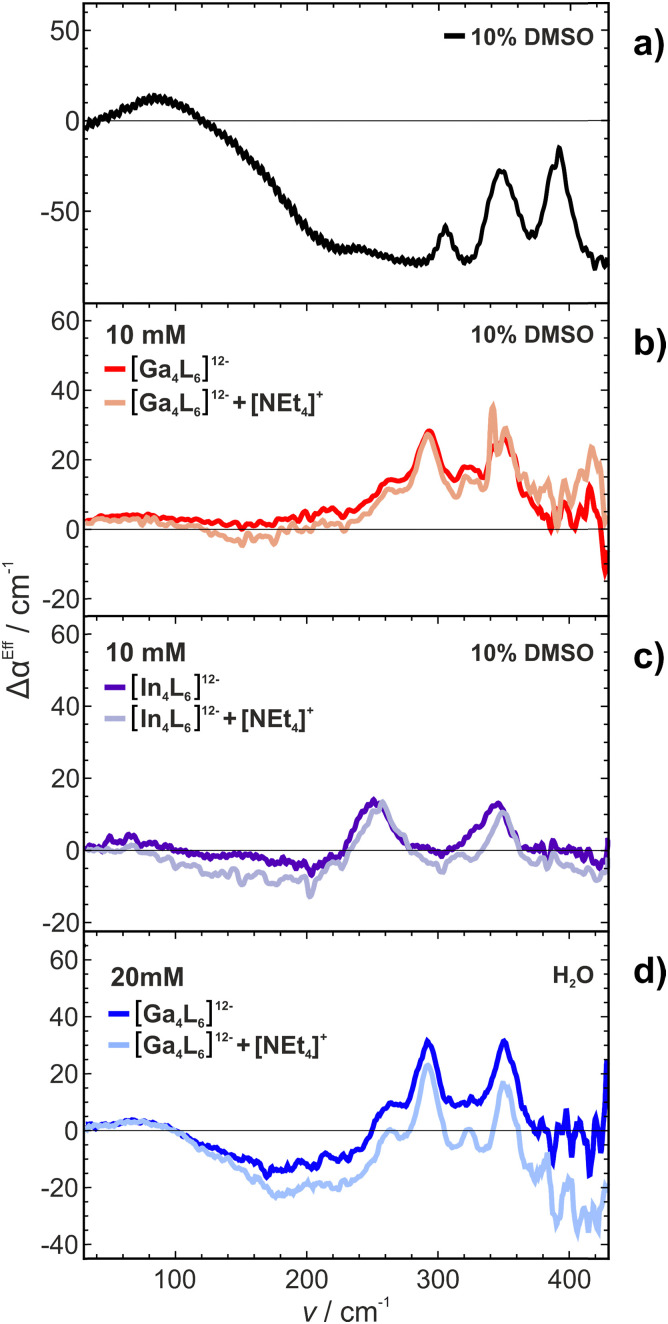
Experimental THz spectra plotting the effective difference absorption coefficients Δ*α*^Eff^ as a function of frequency. (a) Mixture DMSO in water with DMSO : H_2_O (1 : 9, V/V). (b) [Ga_4_L_6_]^12−^ (10 mM) in an aqueous DMSO solution (red) or encapsulated NEt_4_^+^ as guest (light red). (c) [In_4_L_6_]^12−^ (10 mM) in an aqueous DMSO solution (purple) or filled with NEt_4_^+^ as guest (light purple). (d) [Ga_4_L_6_]^12−^ (20 mM) in an aqueous solution (blue) or with encapsulated NEt_4_^+^ as guest (light blue). For individual data sets, see ESI.†

In eqn (1), the spectrum of *α*_10%DMSO_ for 10% DMSO solution was subtracted by a density corrected bulk water spectrum (Fig. S17, light gray, ESI†), while *α*_sample_ for the hosts solvated in 10% DMSO were subtracted by a density corrected spectrum of the 10% DMSO solution (eqn (2) and Fig. S17, black, ESI†). The effective difference absorption coefficients Δ*α*^Eff^ of the solvent (eqn (1)) as well as of the solutes and their hydration water (eqn (2)) were obtained by taking the difference to bulk water and DMSO:1
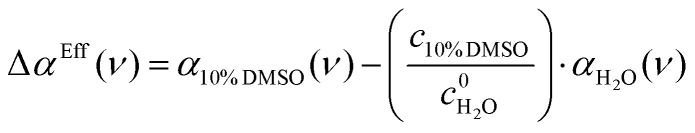
2
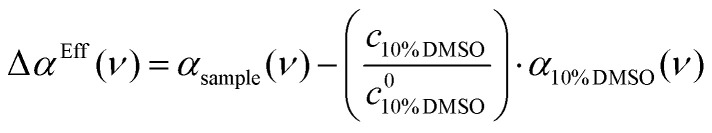
*c*_10%DMSO_ and *c*^0^_10%DMSO_ are the molar concentrations of the solvent in the presence and absence of the solute respectively, while 
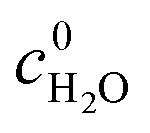
 is the molar concentration of pure bulk water. The obtained spectra of Δ*α*^Eff^ are shown in Fig. 2. Details of the analysis of Δ*α*^Eff^ for [Ga_4_L_6_]^12−^ in water can be found in the ESI† (eqn (S8)). All molar concentrations were deduced from mass density measurements at 20 °C. Thereby we correct for any concentration-dependent change in the apparent molar volume of the solute.^42^

The difference THz spectrum of a 10% mixture of DMSO in water Δ*α*^Eff^_10%DMSO_ compared to bulk water is plotted in Fig. 2a (black). It shows a broad absorption mode in the frequency range between 100 cm^−1^ and 150 cm^−1^. In addition, three sharp intermolecular modes were detected at 310 cm^−1^, 350 cm^−1^ and 390 cm^−1^. Most notable, the latter two were also observed in measurements of pure DMSO (Fig. S17, red, ESI†).

In Fig. 2b (red) and Fig. 2c (purple) we plot the effective difference absorption coefficients Δ*α*^Eff^ of [Ga_4_L_6_]^12−^ and [In_4_L_6_]^12−^ dissolved in aqueous 10% DMSO solutions, while Fig. 2d (blue) shows Δ*α*^Eff^ of [Ga_4_L_6_]^12−^ dissolved in pure water. The spectra are compared in the presence 
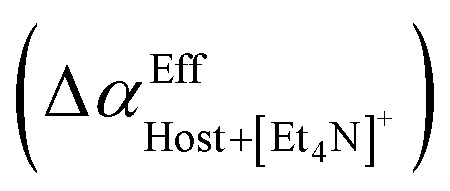
 or absence (Δ*α*^Eff^_Host_) of the cationic guest.

In both cases we observe a decrease in Δ*α*^Eff^ upon encapsulation of NEt_4_^+^ for an aqueous DMSO mixture: in the range between 100 cm^−1^ and 270 cm^−1^ for [Ga_4_L_6_]^12−^ and between 100 cm^−1^ and 200 cm^−1^ for [In_4_L_6_]^12−^, respectively. A direct comparison of the [Ga_4_L_6_]^12−^ nanocages in pure water *vs.* [Ga_4_L_6_]^12−^ nanocages in 10% DMSO aqueous solution shows that the difference in absorption compared to the sample with an encapsulated guest is smaller in case of the DMSO-water mixture, see Fig. 2b and d.

For [In_4_L_6_]^12−^, we observe intramolecular modes of the tetrahedral cage between 250 cm^−1^ and 380 cm^−1^, see Fig. 2c. These intramolecular modes exhibit a broader linewidth compared to the cavity modes of the [Ga_4_L_6_]^12−^ nanocages. We propose that the line broadening of the [In_4_L_6_]^12−^ modes can be rationalized by a more effective coupling of the low frequency cavity modes with the solvent modes than in case of [Ga_4_L_6_]^12−^. This supports previous theoretical predictions.^43^

In the next step of our analysis, we calculate the double-difference absorption spectra 
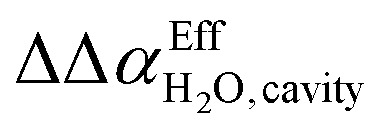
 to obtain information on the impact of the encapsulated solvent. We subtract the THz spectra of the host–guest complex 
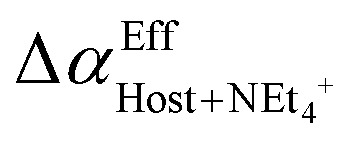
 from the spectrum of the solvated host Δ*α*^Eff^_Host_:4
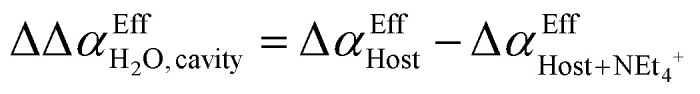


To estimate the number of waters inside the cage, the deduced spectrum was compared to the absorption spectrum of a known number of water molecules in the cavity, for details see ESI.† (ref. 51)

**Fig. 3:**
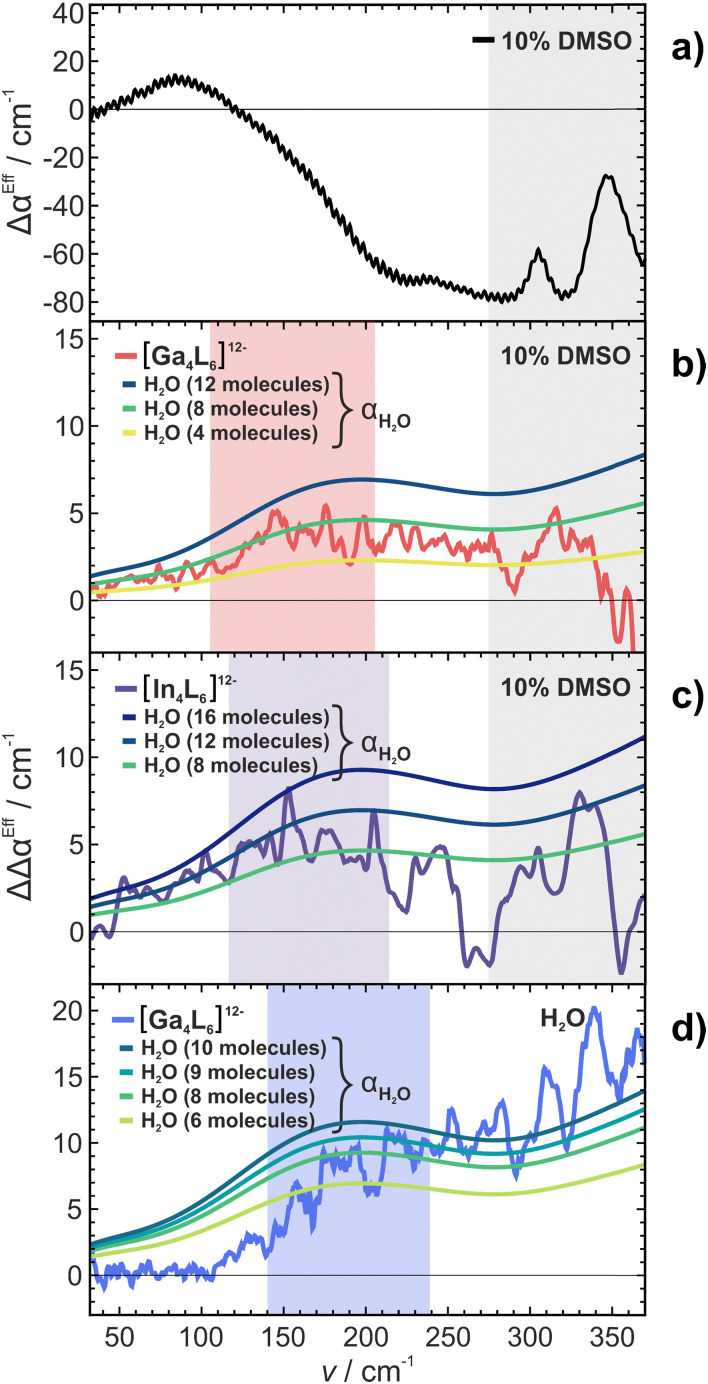
Determination of the effective number of solvent molecules in the nanocage. All Spectra are plotted in the frequency range between 35 cm^−1^ and 370 cm^−1^. (a) The effective difference absorption coefficient Δ*α*^Eff^ of the mixture DMSO : H_2_O (1 : 9, V/V) in respect to water. (b) Effective double difference absorption coefficient ΔΔ*α*^Eff^ for (10 mM) [Ga_4_L_6_]^12−^ dissolved in 10% DMSO as a function of frequency. The light red curve captures any changes due to encapsulation of the guest. This spectrum is compared to the spectra of 4, 8 and 12 bulk water molecules inside these cages. (c) Effective double difference absorption coefficient ΔΔ*α*^Eff^ for (10 mM) [In_4_L_6_]^12−^ dissolved in 10% DMSO as a function of frequency. The purple curve captures any changes due to encapsulation of the guest. This spectrum is compared to the spectra of 8, 12 and 16 bulk water molecules inside these cages. (d) Effective double difference absorption coefficient ΔΔ*α*^Eff^ for (20 mM) [Ga_4_L_6_]^12−^ dissolved in 10% DMSO as a function of frequency. The blue curve captures any changes due to encapsulation of the guest. This spectrum is compared to the spectra of 6, 8, 9 and 10 bulk water molecules inside these cages, as predicted for 20 mM supramolecular cages. The colored regions in light red, light purple and light blue mark the absorption maxima, while the frequency range for DMSO modes are highlighted in light gray. For individual data sets, see ESI.†

Based on Fig. 3b (red) we deduce a number of 8 ± 1 water molecules for [Ga_4_L_6_]^12−^ in 10% DMSO, which can be compared to the number of 9 ± 1 water molecules for [Ga_4_L_6_]^12−^ in pure water shown in Fig. 3d (blue).^51^ Interestingly, the number of water molecules which are impacted by the presence of the [In_4_L_6_]^12−^ cavity is estimated to be 12 ± 1 water molecules for a mixture of 10% DMSO in water, which is higher than number of 8 ± 1 water molecules of [Ga_4_L_6_]^12−^ in a mixture of 10% DMSO and (Fig. 3c, purple).

When we compare Fig. 3b and c, *i.e.* ΔΔ*α*^Eff^ for [Ga_4_L_6_]^12−^ and [In_4_L_6_]^12−^ in 10% DMSO, we find that despite subtraction of the solvent corrected spectra, a weak DMSO mode is still visible (Fig. 3, light gray regions). The specific DMSO-associated features in the 270–370 cm^−1^ range were identified based on comparison to previously observed modes in pure DMSO (Fig. S17, ESI†) and aqueous 10% DMSO solutions (Fig. 3a). After subtraction of the known absorption of the density corrected water/DMSO mixture, weak residual DMSO signals at 300 and 350 cm^−1^ are still visible, see Fig. 3c, which led us to the conclusion of an enrichment of DMSO molecules within the nanocages. This supports our hypothesis that preferentially DMSO interacts with the cavity. Deviations in the lineshape of these bands can be explained by the interaction between the DMSO molecules and the confined water or the coupling of DMSO with the cage and the hydration water mode.

Thus, we conclude that DMSO molecules are enriched in both cavities compared to a bulk 10% DMSO water mixture. We propose that DMSO preferentially occupies the cavity inside the host, for both [Ga_4_L_6_]^12−^ and [In_4_L_6_].^12–32,41–44^

Furthermore, we want to note a redshift for the hydrogen bond stretch of the encapsulated water. The band is shifted from 190 cm^−1^ for bulk water to ∼155 cm^−1^ for [Ga_4_L_6_]^12−^ and ∼165 cm^−1^ for [In_4_L_6_]^12−^ in 10% DMSO (Fig. 3b, light red region and Fig. 3c, light purple region). For comparison, the absorption maxima of encapsulated water in [Ga_4_L_6_]^12−^ dissolved in water was observed at ∼185 cm^−1^ (Fig. 3d, light blue region). The redshift indicates a weakening of the radial H-bond interaction, which can result from a less favorable H-bond structure. Thus, the water structure within the host cavities is affected upon addition of DMSO.

### Guest binding thermodynamics

In addition, we carried out an experimental thermodynamic investigation of guest binding in these mixed solvent conditions. We performed a van’t Hoff analysis using ^1^H NMR to derive the changes in the entropy (Δ*S*) of encapsulation of NEt_4_^+^.

**Fig. 4:**
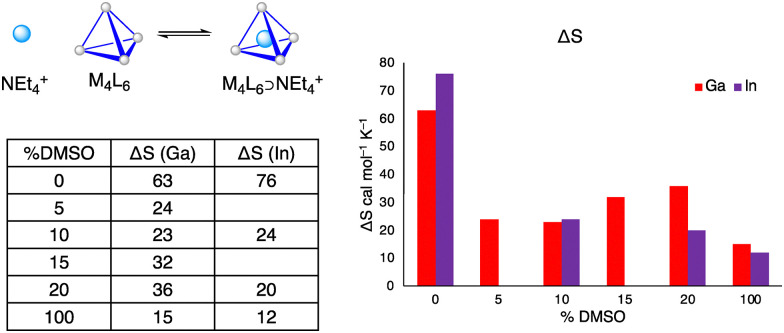
Entropy of encapsulation for NEt_4_^+^ in [Ga_4_L_6_]^12−^ and [In_4_L_6_]^12−^ in cal mol^−1^ K^−1^, respectively, measured by an NMR van’t Hoff technique. The standard error is in range of 3 to 5 cal mol^−1^ K^−1^, see ESI† (Tables S3 and S4).

In Fig. 4, the resulting encapsulation entropy Δ*S* for a NEt_4_^+^ guest is shown as a function of increasing DMSO concentrations in water, for [Ga_4_L_6_]^12−^ and [In_4_L_6_]^12−^ respectively. Most remarkable, for both metal cages, Δ*S* is reduced by a factor of ∼3–4 as soon as DMSO is added as a cosolvent to water (Δ*S*_water_ > Δ*S*_DMSO/water_). The effect is more pronounced in the case of [In_4_L_6_]^12−^ than in the case of [Ga_4_L_6_]^12−^. Both hosts appear to concentrate DMSO within their cavity, which agrees with our THz spectra. For mixtures between 5% to 20% DMSO, similar entropy values were measured for both hosts, likely indicating a saturation of the host cavity with encapsulated DMSO.

Without the addition of DMSO, *i.e.* in pure water, the encapsulation entropy of NEt_4_^+^ is slightly higher for [In_4_L_6_]^12−^ compared to [Ga_4_L_6_]^12−^, *i.e.* the release of encapsulated water is entropically more favorable for [In_4_L_6_]^12−^. While this is consistent with theoretical expectations^43^ and previous experimental reports,^49^ based on our THz experiments we can directly relate this to the higher number of embedded entropically unfavorable water molecules in In cages *versus* Ga cages.

A guest binding experiment was also performed (see ESI†), showing a stronger binding affinity for NEt_4_^+^ in [In_4_L_6_]^12−^ compared to [Ga_4_L_6_]^12−^ by approximately a factor of two (Table S7, ESI†). As the temperature is raised, the affinity for [In_4_L_6_]^12−^ increases. With *T*Δ*S* being the most temperature dependent contribution to the free energy, this experimental result points to a greater entropically favorable (ΔΔ*S* > 0) guest binding in [In_4_L_6_]^12−^ compared to [Ga_4_L_6_]^12−^. Our findings demonstrate that these host–guest interactions, which determine the catalytic efficiency of the cage, can be efficiently tuned both by variation of the solvent composition and by modification of the cage vertices.

### Theoretical analysis of guest binding and host solvation

Here we report the results of accompanying calculations to quantify the change in entropy upon capsulation, *i.e.* the difference when moving the guest from outside to inside the cage thereby displacing the solvent molecules inside and moving them outside.^9,33^ Previously, the encapsulation entropy Δ*S* has been shown to be dominated by the free energy of cavity formation (Δ*μ*_cavity_ ≈ −*T*Δ*S*_cavity_ for cavities with radius *r* < 7 Å, as for our cage): water within the cage exists in a high-energy state, due to confinement and undercoordination.^51^ These changes on solvation entropy inside the cavity provide an entropic driving force for guest encapsulation due to the release of water from the cage to the bulk upon guest binding, which is entropically favorable.^51^ The experimental results, see Fig. 3 and 4 show that this driving force is considerably reduced upon addition of at least 5% of DMSO. Therefore, we relate the observed change in the THz spectra to changes in the solvation entropy Δ*S* (eqn (4)). Previously we found that changes in the spectroscopic observables (the amplitude of the redshifted H-bond stretching mode) probed around 165 cm^−1^ and Δ*S* are linearly correlated.^46,55,56^4
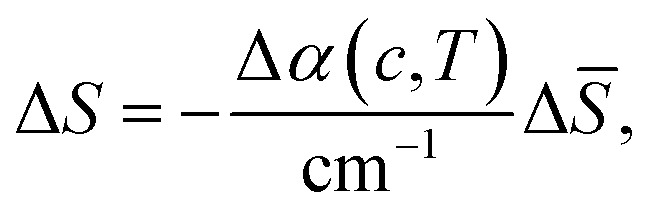
with Δ*S̄* being 4.4 J mol^−1^ K^−1^.

This method holds for aqueous solutions. For the 20 mM [Ga_4_L_6_]^12−^ solution, we estimate an amplitude of ΔΔα of *ca.* 10 cm^−1^. We note that approximately 9 water molecules are encapsulated in each cavity, implying that the amplitude of the encapsulated water corresponds to *ca.* 180 mM encapsulated water molecules. Thus, based on eqn (4), we deduced a value of Δ*S* for the encapsulated water of Δ*S* = 0.18 mol × 10 cm^−1^ × 4.4 J mol^−1^ K^−1^ cm = 7.9 J K^−1^ = 1.9 cal K^−1^. This is in the close to the measured value of Δ*S* = 0.02 mol × 63 cal mol^−1^ K^−1^ = 1.3 cal K^−1^. This indicates that solvation entropy is a major contribution of the total entropy change. However, any change in cage flexibility and electric fields will also have an impact on the total Δ*S*, as probed by calorimetry. However the same method cannot be applied to the THz measurements in a 10% DMSO mixture, since both DMSO and water will contribute to the absorption in the specified frequency range and to changes in entropy. We thus restricted our further analysis to a relative comparison of amplitude for both cages in the same mixture.

We measured the amplitudes for *c* ≈ 10 mM (*T* = 293 K) at ∼155 cm^−1^ for [Ga_4_L_6_]^12−^ in 10% DMSO and ∼165 cm^−1^ for [In_4_L_6_]^12−^ in 10% DMSO (Fig. 3b and c). ΔΔ*α* is the difference between Δ*α* of the cage with encapsulated solvent and the cage with encapsulated guest. The difference ΔΔ*α* reports on changes compared to 10% DMSO solution. The observed mode at ∼165 cm^−1^ shows similarity to a two-dimensional H-bond network. This band reports on the cavity formation.^56^ Here we propose that, due to the confinement, undercoordination and local electric fields, the encapsulated water is destabilized with respect to water solvating hydrophobic solutes, as detailed previously.^51^

Comparison of the amplitude of ΔΔ*α*, [In_4_L_6_]^12−^ shows an increased value compared to [Ga_4_L_6_]^12−^. This is in line with the relative increase when comparing the Δ*S* values observed in van't Hoff analysis for [In_4_L_6_]^12−^ compared to [Ga_4_L_6_]^12−^ (see Fig. 4). These observations suggest that the differences in encapsulation entropy between the two hosts arise in part from the contribution of released water.

**Fig. 5:**
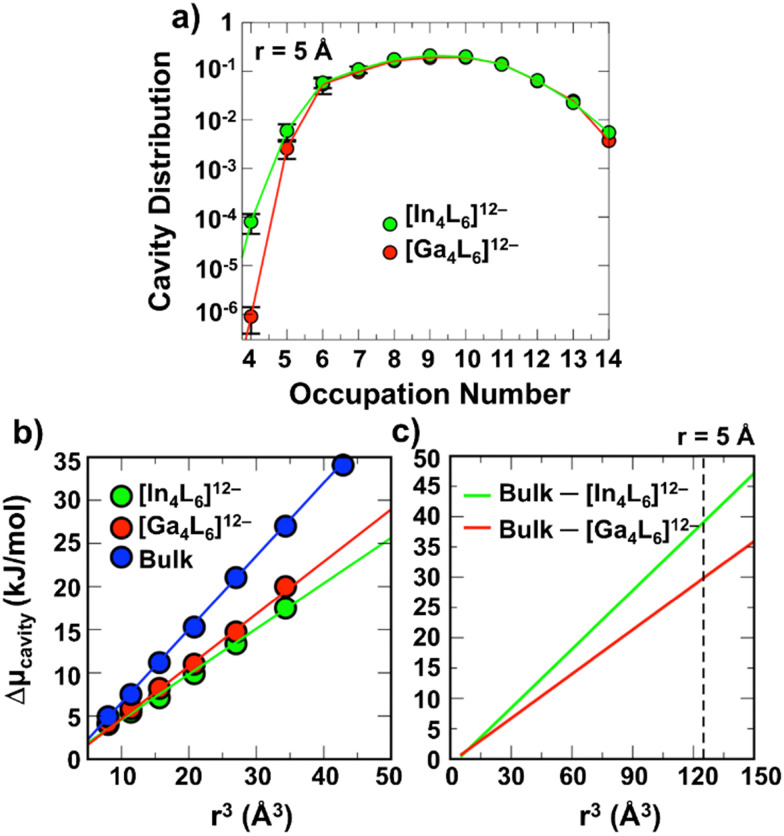
Cavity distributions and cavitation free energies for water in a sphere radius of 5.0 Å within the supramolecular cage. (a) The computed occupancy data for [In_4_L_6_]^12−^ (green) and [Ga_4_L_6_]^12−^ (red). Error bars have been estimated by evaluating the standard deviation of the occupancy numbers within independent parts of the MD trajectories. Note that for some points the error bars fall within the symbol. (b) Cavitation free energy (Δ*μ*_cavity_) inside the [In_4_L_6_]^12−^ (green) and [Ga_4_L_6_]^12−^ (red) compared to bulk (blue) as a function of the cavity volume. Symbols represent the calculated Δ*μ*_cavity_ values for *r* = 2.0, 2.25, 2.5, 2.75, 3.0, 3.25 Å, while the solid lines are linear fits. The error bars fall within the circles. (c) Difference between Δ*μ*_cavity_ in the bulk and inside the cage as a function of cavity size, obtained by subtracting the red or green to the blue curve in (b). The linear trend of Δ*μ*_cavity_*vs*. cavity volume allows to extrapolate to *r* = 5 Å (vertical dashed line, corresponding to the cavity formed by common guests). Values of 39.1 ± 2.5 kJ mol^−1^ and 29.8 ± 2.5 kJ mol^−1^ are obtained from the extrapolation for [In_4_L_6_]^12−^ and [Ga_4_L_6_]^12−^ respectively.

To further validate this interpretation, DFT-MD simulations^51^ were used for both [In_4_L_6_]^12−^ and [Ga_4_L_6_]^12−^. The average number of confined water molecules was evaluated over the equilibrated portion of the trajectory and found to be 12.0 ± 0.9 for the In-cage and 12.4 ± 0.7 for the Ga-cage. In both cases, 8–10 water molecules exhibit long residence times within the cavity, persisting for over 95% of the trajectory, while the remaining water molecules undergo fast exchange with the bulk as seen in the cavity distributions in Fig. 5a. To provide a better estimate of the free energy of cavity formation for the two metal nanocages, we provide a rough free energy analysis. Given the prohibitive cost of DFT-MD for more standard free energy perturbation methods, we chose instead to provide an estimate of the chemical potential *via* cavity statistics. (10.1073/pnas.93.17.8951) Calculations using straightforward equilibrium simulations, as is done here, should provide sufficient estimates for smaller volumes with corresponding values of Pv(0) < 10^−8^ which is thus adequate for this study. (10.1073/pnas.93.17.8951 and 10.1007/s10955-011-0269-9) Fig. 5b shows that the cavitation free energy, Δ*μ*_cavity_, scales linearly with the small volume for both [In_4_L_6_]^12−^ and [Ga_4_L_6_]^12−^, and as the cavity gets larger, both [In_4_L_6_]^12−^ and [Ga_4_L_6_]^12−^ have a reduced slope, and hence greater propensity for cavity formation with respect to the bulk liquid. The linear trend of the difference between the nanocage and bulk solvent *vs*. cavity volume allows extrapolation to *r* = 5 Å, corresponding to the cavity formed by common guests. As seen in Fig. 5c, the formation of a 5 Å cavity is shown to be favoured with respect to bulk solvent by ∼30 ± 2.5 kJ mol^−1^ for [Ga_4_L_6_]^12−^ and 39 ± 2.5 kJ mol^−1^ for [In_4_L_6_]^12−^.

Based on these results we can explain, why the [M_4_L_6_]^12−^ cages can efficiently encapsulate not only positively charged guests (favored by the electrostatic interaction term) but also neutral hydrophobic/amphiphilic molecules.^44^ While electrostatic interactions are expected to contribute mainly to enthalpic contributions, which likely dominate for water, the cavitation entropic driving force does not only depend on the cavity ligand, but the solvent mixture is decisive. In order to quantify the solvation contribution to guest encapsulation upon addition of DMSO in water we have to take into account the volume exclusion by DMSO. Notably, the first-order entropy change is proportional to the volume of water released from the cage cavity into the bulk and is considered as a function of the cavity volume (see in Fig. 5c and ESI†). Based on this model we estimate the expected change in Δ*μ*_cavity_ upon reduction of the cavity volume. Considering that a DMSO molecule forms a cavity of ∼*r* = 4.8 Å (as given by the distance between the geometric center of DMSO and the water oxygen centers in the 1st solvation shell), we calculate Δ*μ*_cavity_ (DMSO) = 34.5 kJ mol^−1^ for [Ga_4_L_6_]^12−^. This exceeds the value for pure water implying that DMSO molecules will be preferentially located within the cage.

The amount of DMSO that can be encapsulated *n*_DMSO_(max) is mostly limited by steric effects. Considering that the radius inside the cage is of ∼*r* = 5–6.0 Å, we assume that not more than one DMSO molecule can be encapsulated in each cage, *i.e. n*_DMSO_(max) = 1, independent of the solvent mixture. For a given bulk DMSO concentration *n*^0^_DMSO_, *n*_DMSO_ is deduced from Δ*μ*_cavity_ according to a conditioned (by n_DMSO_(max) = 1) Boltzmann probability:5
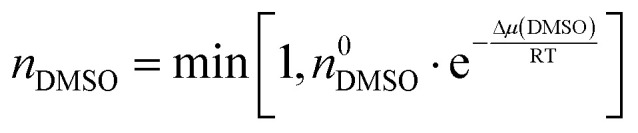


From eqn (5), we find that even for a partial contribution of 5% DMSO all cages must be filled with a single DMSO molecule. This stays constant, even for higher concentrations since it is restricted by steric constraints. If DMSO is present inside the cage before guest encapsulation, the water volume released upon guest binding is reduced according to:

6 *V*_effective_ = *V*_cage_ − *n*_DMSO_·*V*_DMSO_

Therefore, the Δ*μ*_cavity_ entropic gain upon guest binding is expected to be reduced.

**Fig. 6:**
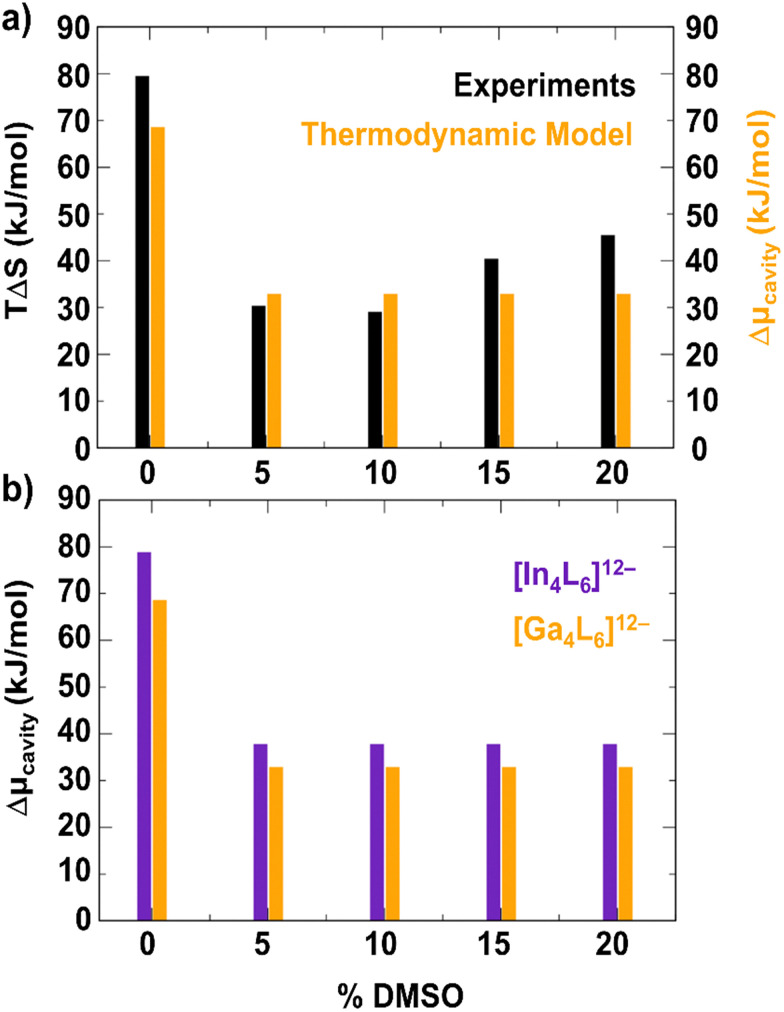
Results of the thermodynamic model. (a) The cavitation free energy contribution to encapsulation in [Ga_4_L_6_]^12−^ calculated from DFT-MD with the thermodynamic model described in the text (orange, Δ*μ*_cavity_ ≈ −*T*Δ*S*_cavity_) is compared to the experimental results from van’t Hoff analysis (same as in Fig. 4 but plotted as -*T*Δ*S*). (b) Comparison between theoretical results on encapsulation Δ*μ*_cavity_ between [In_4_L_6_]^12−^ and [Ga_4_L_6_]^12−^.

The results of such a thermodynamic model are plotted in Fig. 6 and compared to the experimental results of the van’t Hoff analysis in Fig. 4 for the [Ga_4_L_6_]^12−^. As can be seen in Fig. 6a, the results of the simplified thermodynamic model are in good agreement with the experimentally observed reduction in Δ*S* from the van’t Hoff analysis upon addition of 5% DMSO. The model also explains now why Δ*S* is not further decreased when more DMSO is added. While the dramatic decrease by more than a factor of two upon addition of 5% DMSO is well reproduced, any smaller changes are due to the simplifications made: not considering the changes of the encapsulated water structure compared to bulk water.^57^

In Fig. 6b, we compare the theoretical result for [In_4_L_6_]^12−^*vs*. [Ga_4_L_6_]^12−^. The model indicates a larger encapsulation Δ*μ*_cavity_ ≈ −*T*Δ*S*_cavity_ for [In_4_L_6_]^12−^ than for [Ga_4_L_6_]^12−^, in agreement with the experimental results. However, both decrease significantly upon addition of 5% DMSO. Our model rationalizes this: the overall larger Δ*μ*_cavity_ for [In_4_L_6_]^12−^ is due to a slightly stronger destabilization of encapsulated water within the host compared to the [Ga_4_L_6_]^12−^ (see linear fits in Fig. 5). The decrease in Δ*S* upon addition of 5% DMSO is due to the fact that in both cases a single DMSO is preferentially residing inside the cage, thereby reducing the volume of released water upon guest binding for both systems.

## Discussion and conclusion

In summary, by a joint experimental and theoretical study of [Ga_4_L_6_]^12−^ and [In_4_L_6_]^12−^ in a solvent mixture by THz spectroscopy, DFT-MD simulations and NMR experiments we investigated the differences in guest encapsulation between the two hosts. While [In_4_L_6_]^12−^ displays slightly higher hydrophobicity and lower solubility in water compared to that of [Ga_4_L_6_]^12−^, [In_4_L_6_]^12−^ encapsulates more water molecules due to mostly enthalpic effects in which [In_4_L_6_]^12−^ has a stronger binding affinity for larger cationic guests such as NEt_4_^+^, as well as a modestly higher entropic gain when displacing water from its cavity.

Indium's weaker acidity promotes stronger M–O coupling with water molecules, resulting in weaker In–O bonds compared to Ga–O bonds.^43,48^ This leads to higher basicity of the catecholates, facilitating water molecule ordering inside and outside the host cavity. The stronger interactions between indium-based vertices and water molecules may outweigh entropic costs, aligning with observed enthalpy-entropy compensation effects in previous van’t Hoff studies of these host systems.^37–40,51^ This trend of weaker M–O bonds at the host vertices correlating to higher entropy of encapsulation has been demonstrated in a study^49^ with a wide scope of host structures, including Si(iv), Ge(iv), Fe(iii), along with Ga(iii) and In(iii), suggesting that the strength of the M–O bonding in the host influences its solvation and subsequently guest binding thermodynamics.

In line with these expectations, we determined an entropic driving force for guest encapsulation due to the release of encapsulated water upon guest binding which is increased from 63 to 76 cal mol^−1^ K^−1^ for [In_4_L_6_]^12−^ compared to [Ga_4_L_6_]^12−^. Surprisingly, we observed a much more significant change in the entropy change upon guest encapsulation upon addition of 10% DMSO into the aqueous solution: in this case Δ*S* was reduced from 63 to 23 cal mol^−1^ K^−1^ for [Ga_4_L_6_]^12−^ and from 76 to 24 cal mol^−1^ K^−1^ for [In_4_L_6_]^12−^. We determine that the experimentally observed preferred encapsulation of DMSO reduces the volume of released water upon guest binding. Finally, the encapsulation entropies obtained from van’t Hoff analyses are in good agreement with the values estimated from the thermodynamic model.

A further increase in DMSO beyond 5% does almost not affect Δ*S*, since only a single DMSO molecule can be encapsulated independent of the mixture. Hence, the local solvent composition within the cage is found to be a crucial, most sensitive parameter in regulating guest binding affinity. Our study poses the solvent composition can be used to effectively tune guest–binding interactions, independent of the exchange of the metal ligands. Our results showing the preferential encapsulation of DMSO in the nanocage and the significant impact on the free energy change upon guest encapsulation not only provide insight into host–guest binding interactions but can be leveraged in potential applications in chemical separations, cargo-transport and reaction steering by *ad-hoc* tuning of solvent compositions.

## Materials and methods

[Ga_4_L_6_]^12−^ and [In_4_L_6_]^12−^ were synthesized according to a modified version of a previously reported procedure.^53^

For standard calorimetric measurements, samples containing one equivalent of [M_4_L_6_]^12−^ host and one equivalent of NEt_4_^+^ guest were heated, and the relative molar ratios of the encapsulated *versus* exterior guest, as determined by ^1^H NMR, were recorded and used to calculate the equilibrium constant (*K*_eq_) at different temperatures:

7 −*R* ln(*K*_eq_) = Δ*H* − *T*·Δ*S*

With the ideal gas constant *R* and temperature *T*, −*R* ln(*K*_eq_) can then be plotted against 1/*T* (in Kelvin), and a linear function can be fitted to deduce the slope Δ*H* and the intercept Δ*S*.

The 1 : 1 binding with NEt_4_^+^ is verified by 1 H-NMR of the synthesized host, which shows encapsulated NEt_4_^+^ and no free salt in solution (see ESI† for details about the sample preparation, measurements, and data analysis).

### THz spectroscopy

Far-infrared (FTIR) absorption spectroscopy was utilized to record spectra of supramolecular gallium and indium hosts in 10 mM aqueous solution at 293 K and in the 30–450 cm^−1^ frequency range using a Bruker Vertex 80v Fourier Transform Infrared Spectrometer equipped with a liquid helium cooled bolometer (detector) from Infrared Laboratories. The sample solutions were placed in a temperature-controlled liquid transmission cell with polycrystalline diamond windows and a 25-μm-thick Kapton spacer. Each spectrum was generated by averaging 128 scans at a resolution of 2 cm^−1^. The resulting double-difference absorption spectra were then smoothed with a 2 cm^−1^ moving average over five points.

### AIMD

All calculations were performed as previously reported^51^ with DFT using the dispersion corrected *meta*-GGA functional B97M-rV^58–60^ in combination with a DZVP basis set optimized for multigrid integration^61^ as implemented in the CP2K software package.^62,63^ A cubic box of 30 Å represented the simulated system of 2572 atoms (including 760 water molecules) and periodic boundary conditions, five grids, and a cutoff of 400 Ry were used. Three independent AIMD simulations were performed for 30 ps in the NVE ensemble after an equilibration period of 6 ps (3 ps in the NVT ensemble with *T* = 300 K followed by 3 ps in the NVE ensemble).

The cavitation free energy was estimated from MD simulations by calculating the probability, Pv(0), to observe 0 water molecules in a probe volume, *v*, as follows:

8 Pv (0) = exp(−*β*Δ*μ*cav), where *β* = 1 = *k*_B_*T*

## Author contributions

F. D. T., K. N. R., T. H. -G., and M. H. designed research; M. N., K. T. X., S. P. and W. -L. L. performed research; K. T. X., S. P., W. -L. L., G. S., R. G. B., K. N. R., and F. D. T. contributed new reagents/analytic tools; M. N., K. T. X., W. -L. L., G. S., K. N. R., and F. D. T. contributed data and insights; M. N., K. T. X., S. P., W. -L. L., F. D. T., T. H. -G., and M. H. analysed data; M. N., K. T. X., S. P., T. H. G., and M. H. wrote the paper; and M. N., K. T. X., S. P., and W. -L. L. created figures.

## Data availability

The data supporting this article have been included as part of the ESI.†

## Conflicts of interest

There are no conflicts to declare.

## Supplementary Material

CP-027-D5CP00661A-s001
