# Supplementary material for: Tuning the free energy of host–guest encapsulation by cosolvent
Source: Phys Chem Chem Phys. 2025 Apr 22;27(19):10120–8. doi: 10.1039/d5cp00661a (PMC12042075; doi:10.1039/d5cp00661a)
Supplement: CP-027-D5CP00661A-s001 [file CP-027-D5CP00661A-s001.pdf]

Supplementary Information (SI) for Physical Chemistry Chemical Physics.
This journal is © the Owner Societies 2025

Supporting Information for

Tuning the Free Energy of Host-Guest Encapsulation by Cosolvent

Melinda Nolten[1],[a] Kay T. Xia[1],[b,c] Simone Pezzotti,[a] Gerhard Schwaab,[a] Robert G. Bergman,[b,c*] Kenneth N. Raymond,[b,c*] F. Dean Toste,[b,3*] Teresa Head-Gordon,[b-d*] Wan-Lu Li,[e*] Martina Havenith[a*]

[a]Department of Physical Chemistry II, Ruhr University Bochum, 44801 Bochum, Germany

[b]Chemical Sciences Division, Lawrence Berkeley National Laboratory, Berkeley, CA 94720, USA

[c]Department of Chemistry, University of California, Berkeley, CA 94720, USA

[d]Kenneth S. Pitzer Theory Center and Departments of Bioengineering and Chemical and Biomolecular Engineering, University of California, Berkeley, CA 94720, USA

[e]Department of Chemical and Nano Engineering, University of California, San Diego, CA 92093, USA

***Corresponding authors:** Robert G. Bergman, Kenneth N. Raymond, F. Dean Toste, Teresa Head-Gordon, Wan-Lu Li, Martina Havenith

Emails: rbergman@berkeley.edu, raymond@socrates.berkeley.edu, fdtoste@berkeley.edu, thg@berkeley.edu, wal019@ucsd.edu, martina.havenith@rub.de.

This PDF file includes:

Supporting text
Figures S1 to S23
Tables S1 to S8
SI References

[1] These authors contributed equally

General Methods

Unless otherwise noted, all reactions were carried out in oven-dried glassware sealed with rubber septa under a nitrogen atmosphere with Teflon-coated magnetic stir bars. Deuterated solvents were purchased from Cambridge Isotope Laboratories. Reagents were purchased from commercial sources and used without further purification unless otherwise noted.

Proton nuclear magnetic resonance (^{1}H NMR) spectra were taken with AV-300, AVB-400, AVQ-400, AV-500, or AV-600 Bruker spectrometers operating at 300 MHz, 400 MHz, 500 MHz, or 600 MHz. Chemical shifts are reported in parts per million (ppm) with reference to the appropriate residual solvent signal. ^{1}H NMR: $CDCl_3$ (δ: 7.26 ppm), DMSO-d_6 (δ: 2.50 ppm), MeOD (δ: 3.31 ppm), D_2O (δ: 4.79 ppm).(1) ^{1}H NMR multiplicities are reported as follows: s (singlet), d (doublet), t (triplet), q (quartet), sept (septet), m (multiplet).

THz-Far Infrared (THz-FIR) Spectroscopy was performed with a Bruker Vertex 80v Fourier Transform (FTIR) Spectrometer and a silicon bolometer as a detector from Infrared Laboratories. The solvent dimethyl sulfoxide (DMSO, 99.9 %) was purchased from Thermo Fisher Scientific, while ultrapure water was obtained by a water treatment system (ASTM I, TKA Wasseraufbereitungssysteme GmbH). Unless otherwise noted, all supramolecular cavities and guest molecules were provided by Dean Toste's group.

Synthetic Methods

Ga_4L_6 and In_4L_6 were synthesized according to a modified version of a previously reported procedure.(2)

Synthesis of K12Ga4L6

In a 500 mL three-neck round bottom flask equipped with a 500 mL addition funnel, ligand L (5 g, 11.6 mmol, 6 equiv.) and $Ga(acac)_3$ (2.84 g, 7.7 mmol, 4 equiv.) were combined in degassed MeOH (200 mL). This suspension was further sparged with N_2 for 20 minutes. In the meantime, ether (200 mL) was added to the addition funnel and was sparged with N_2 for 30 minutes. KOH (1.95 g, 34.8 mmol, 18 equiv.) was added dropwise as a 1 M solution in degassed MeOH. The milky white solution became homogeneous upon addition of base, and the resulting yellow solution was stirred under N_2 for thirty minutes. Ether was then added dropwise via the addition funnel. Upon the first signs of precipitation, the addition was halted, and the solution was stirred for an additional two hours to allow the slow precipitation of $K_{12}Ga_4L_6$. $K_{12}Ga_4L_6$ was isolated by filtration as a pale-yellow solid, dried briefly under vacuum, and immediately transferred to an air free glovebox. ^{1}H NMR (500 MHz, Methanol-*d*4) δ 8.05 (d, J = 7.8 Hz, 12H), 7.77 (d, J = 8.6 Hz, 12H), 7.25 (d, J = 8.2 Hz, 12H), 6.96 (t, J = 8.2 Hz, 12H), 6.66 (dd, J = 7.3, 1.6 Hz, 12H), 6.37 (t, J = 7.8 Hz, 12H).

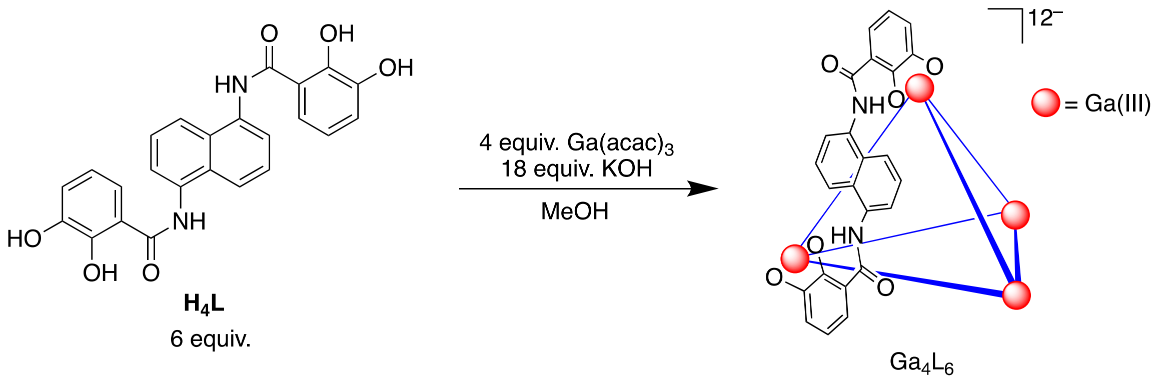

Synthesis of $K_{12}In_4L_6$

In an air-free glove box, ligand L (300 mg, 0.70 mmol, 6 equiv.), NMe_4Br (215 mg, 1.40 mmol, 12 equiv.), and KOH (120 mg, 2.14 mmol, 18 equiv.) were added to a 100 mL round bottom flask and then stirred at 720 rpm in MeOH (50 mL) until homogenous. To this solution, $In(acac)_3$ (195 mg, 0.47 mmol, 4 equiv.) was added, and the mixture was stirred at room temperature overnight, upon which a precipitate formed. The host-guest complex $NMe_4^+ \subset In_4L_6$ was isolated by filtration as a pale-yellow solid.
In an air-free glovebox, $NMe_4^+ \subset In_4L_6$ was added as a suspension in a saturated solution of KPF_6 in acetone (200 mL) and MeOH (5 mL) in a 250 mL round bottom flask. This mixture was stirred overnight at 720 rpm at room temperature. The solids were collected by filtration and washed with acetone (20 mL). The guest exchange procedure was repeated three times to generate $K_{12}In_4L_6$ with < 5 % encapsulated NMe_4^+. ^1H NMR (400 MHz, DMSO-d_6) δ 13.56 (s, 1H), 8.04 (d, J = 7.7 Hz, 1H), 7.60 (d, J = 8.7 Hz, 1H), 7.06 (d, J = 7.9 Hz, 1H), 6.91 (t, J = 8.0 Hz, 1H), 6.37 (d, J = 6.6 Hz, 1H), 6.18 (t, J = 7.7 Hz, 1H).

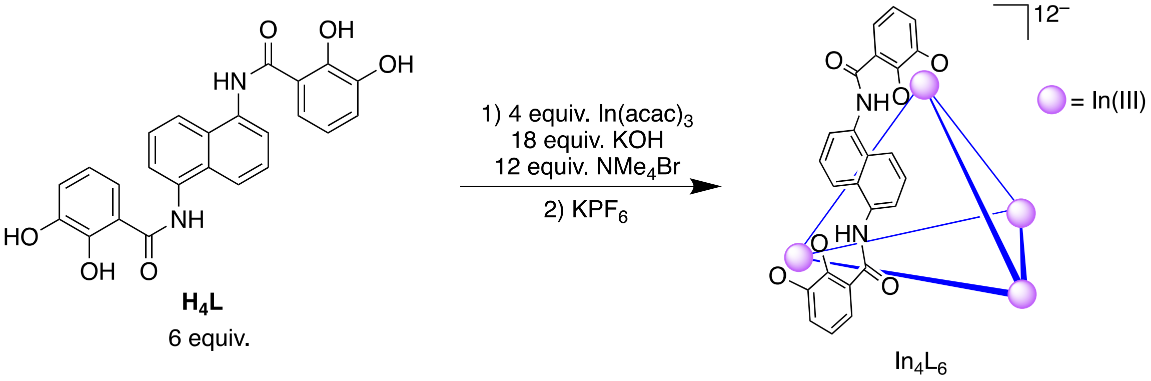

NMR Methods

Method for NMR Experiments

Van't Hoff experiments were conducted to determine the thermodynamic parameters for guest binding in different solvent mixtures.

The method for determining enthalpy and entropy by NMR is as follows:

Sodium tosylate was used as an internal standard. Stock solutions of the appropriate species (Ga_4L_6, In_4L_6, and NEt_4Cl) were prepared in a stock solution containing the internal standard and the appropriate solvent mixture of D_2O and DMSO-d_6. The reaction was monitored immediately by ^1H NMR at a temperature of 25 °C. The concentration of the encapsulated guest was monitored using 8 scans with a delay time of 4 seconds and a 90° pulse of 13.5 μsec for each time point. Data points were taken at increasing temperature intervals of 5 °C, allowing the sample to equilibrate for 15 min at each temperature until no further change in guest concentration was observed. The thermodynamic data were plotted on a van't Hoff graph with the observed equilibria constants vs. 1/T and the data was fit using a linear least squares regression.

NMR Experiments

Van't Hoff Analyses

Measurements were performed with 4 mM M_4L_6, 4 mM NEt_4Cl:

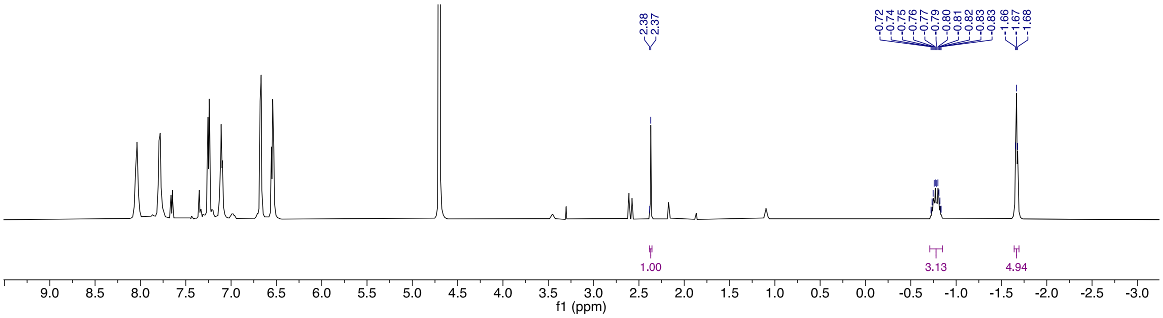

Fig. S1. Example of an NMR spectrum for van't Hoff analysis.

Peak at -0.78 ppm (NEt_4^+, encapsulated, 8H) is integrated against internal standard peak at 2.37 ppm (NaOTs, 3H) and divided by host concentration (determined by host signals between 6.5 – 8.1 ppm) to determine K_{eq} (the equilibrium constant). Using the equation:

$$-Rln(K_{eq}) = \Delta H - T\Delta S \quad \text{(S1)}$$

where R is the ideal gas constant and T is temperature, R*ln*(K_{eq}) is plotted against 1/T (in Kelvin) to obtain a line for which the slope is ΔH and the intercept is ΔS.

The following tables contain data from the van't Hoff experiments for guest binding for **$[Ga_4L_6]^{12-}$** and $[In_4L_6]^{12-}$. Van't Hoff experiments were conducted in duplicate. Standard error is displayed, computed from the duplicate runs. Solvent mixtures are described in volume percentages.

Table S1. Equilibrium constants K_{eq} of $[Ga_4L_6]^{12-}$ and NEt_4^+ at different percentages of DMSO-d_6 at temperatures T.

% DMSO in water	**T** [K]	$\mathbf{K_{eq}}$ [M^{-1}]	% DMSO in water	**T** [K]	$\mathbf{K_{eq}}$ [M^{-1}]
100% DMSO	298	0.81(5)	10% DMSO	298	3.1(2)
	303	0.85(5)		303	3.7(2)
	308	0.99(5)		308	4.6(2)
	313	1.03(5)		313	5.4(2)
	318	1.11(6)		318	6.1(3)
	323	1.49(7)		323	7.2(3)
	328	1.63(8)		328	7.8(3)
20% DMSO	298	4.3(2)	5% DMSO	298	2.6(1)
	303	5.8(2)		303	3.6(2)
	308	8.7(3)		308	3.9(2)
	313	10.6(3)		313	4.6(2)
	318	12.7(3)		318	5.9(3)
	323	18.7(4)		323	6.5(3)
	328	18.1(4)		328	7.4(3)
15% DMSO	298	6.7(2)	0% DMSO	298	25.2(5)
	303	7.7(3)		303	54.1(9)
	308	12.1(3)		308	71(1)
	313	16.0(4)		313	125(2)
	318	17.7(4)		318	216(3)
	323	20.5(5)		323	226(4)
	328	23.1(5)		328	378(5)

Table S2. Equilibrium constants K_{eq} of $[In_4L_6]^{12-}$ and NEt_4^+ at different percentages of DMSO-d_6 at temperatures T.

% DMSO in water	**T** [K]	$\mathbf{K_{eq}}$ [M^{-1}]	% DMSO in water	**T** [K]	$\mathbf{K_{eq}}$ [M^{-1}]
100% DMSO	298	4.2(2)	10% DMSO	298	3.1(2)
	303	4.5(2)		303	4.5(2)
	308	4.8(2)		308	5.3(2)
	313	5.1(2)		313	5.3(2)
	318	5.2(2)		318	6.8(3)
	323	6.0(3)		323	9.0(3)
	328	6.6(3)		328	9.4(3)
20% DMSO	298	5.1(2)	0% DMSO	298	10.2(3)
	303	5.6(2)		303	13.8(4)
	308	6.4(3)		308	17.3(4)
	313	7.5(3)		313	30.1(8)
	318	9.5(3)		318	38.2(9)
	323	10.6(3)		323	139(2)
	328	10.8(3)		328	341(4)

Van't Hoff plots of the data in Tables S1 and S2 are shown below. Error bars for the data points are smaller than the data markers and are omitted, but can be computed from the data tabulated above.

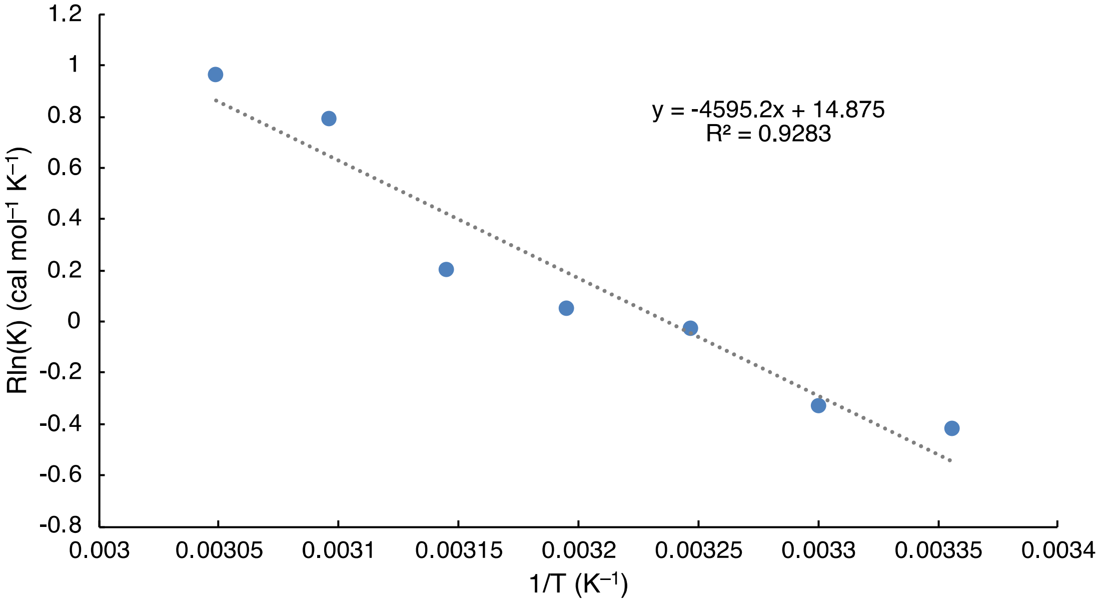

Figure S2: van't Hoff plot of $[Ga_4L_6]^{12-}$ and NEt_4^+ in DMSO-d_6.

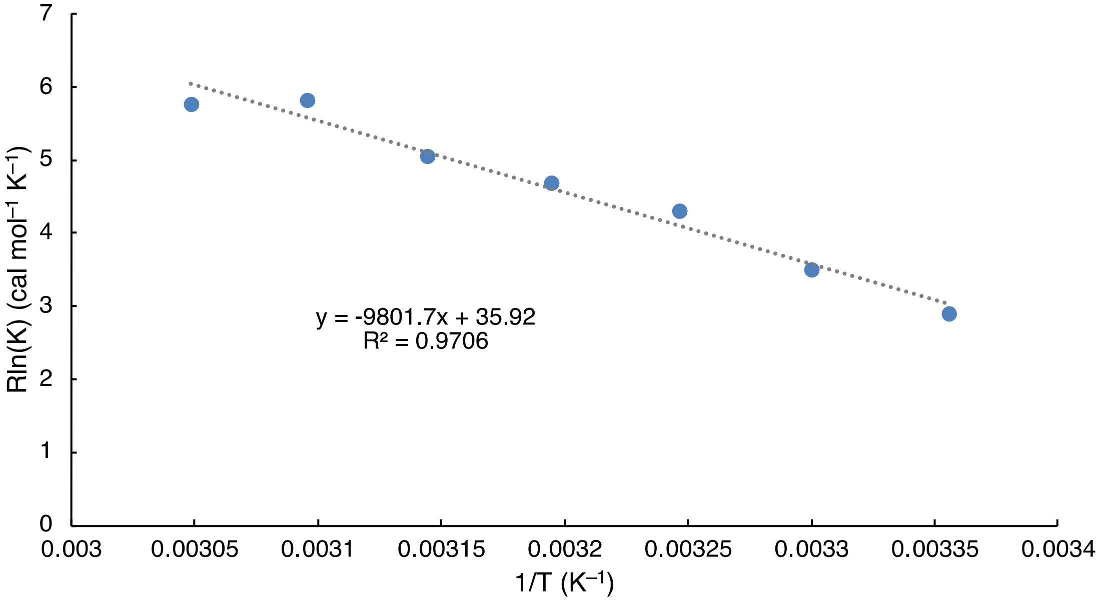

Figure S3: van't Hoff plot of $[Ga_4L_6]^{12-}$ and NEt_4^+ in 20% DMSO-d_6 in D_2O.

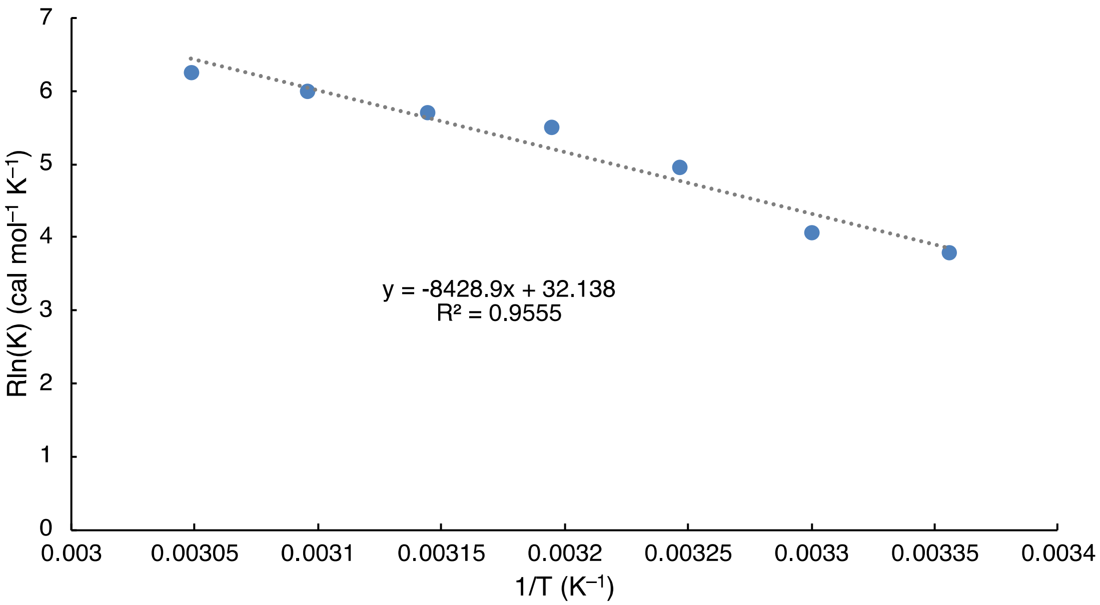

Figure S4: van't Hoff plot of $[Ga_4L_6]^{12-}$ and NEt_4^+ in 15% DMSO-d_6 in D_2O.

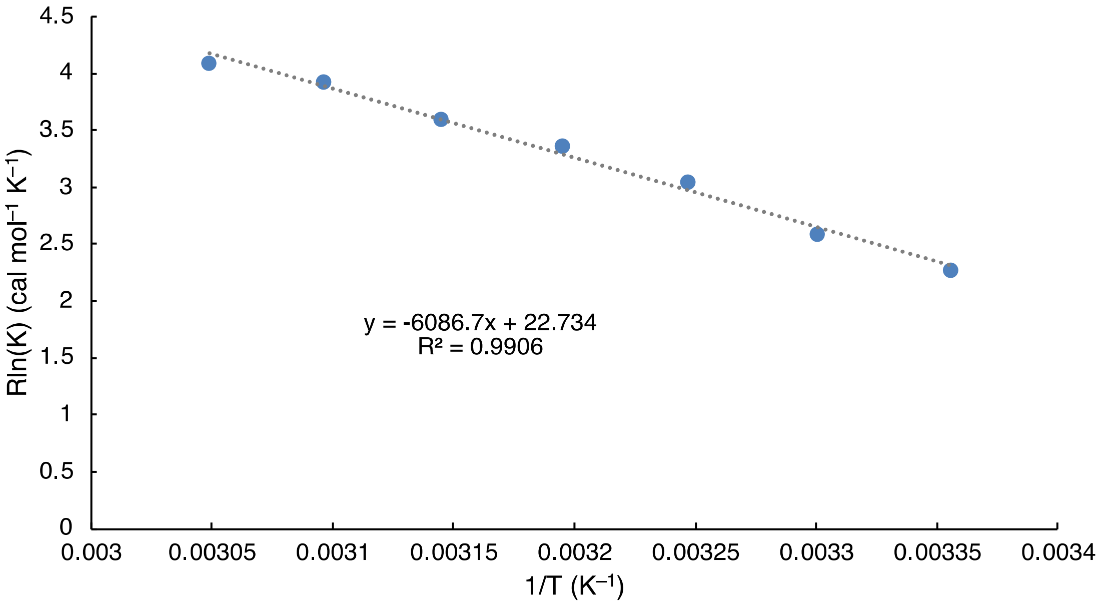

Figure S5: van't Hoff plot of $[Ga_4L_6]^{12-}$ and NEt_4^+ in 10% DMSO-d_6 in D_2O.

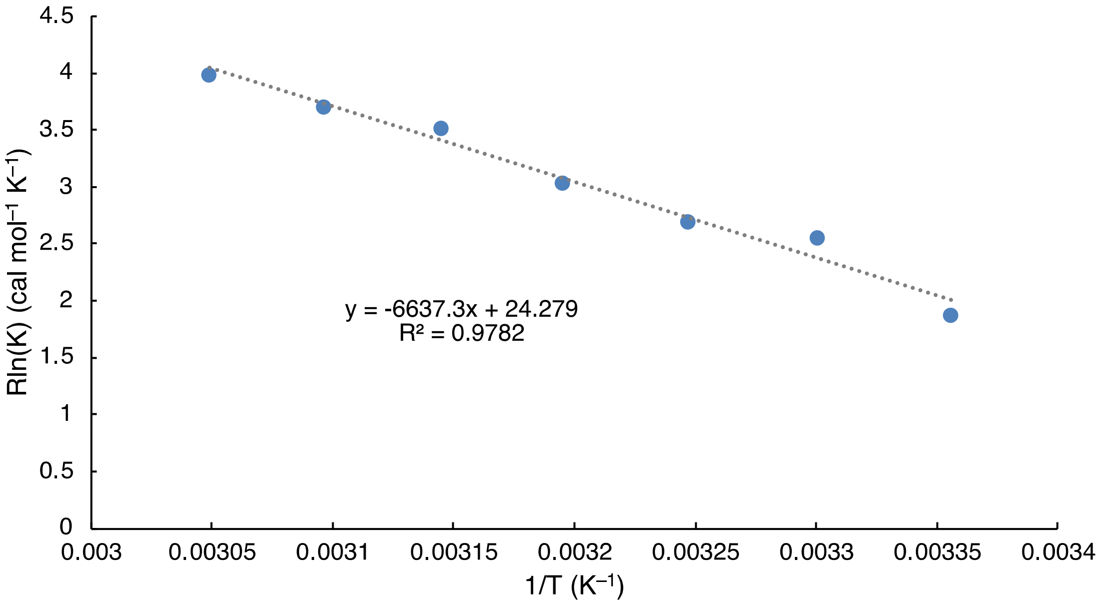

Figure S6: van't Hoff plot of $[Ga_4L_6]^{12-}$ and NEt_4^+ in 5% DMSO-d_6 in D_2O.

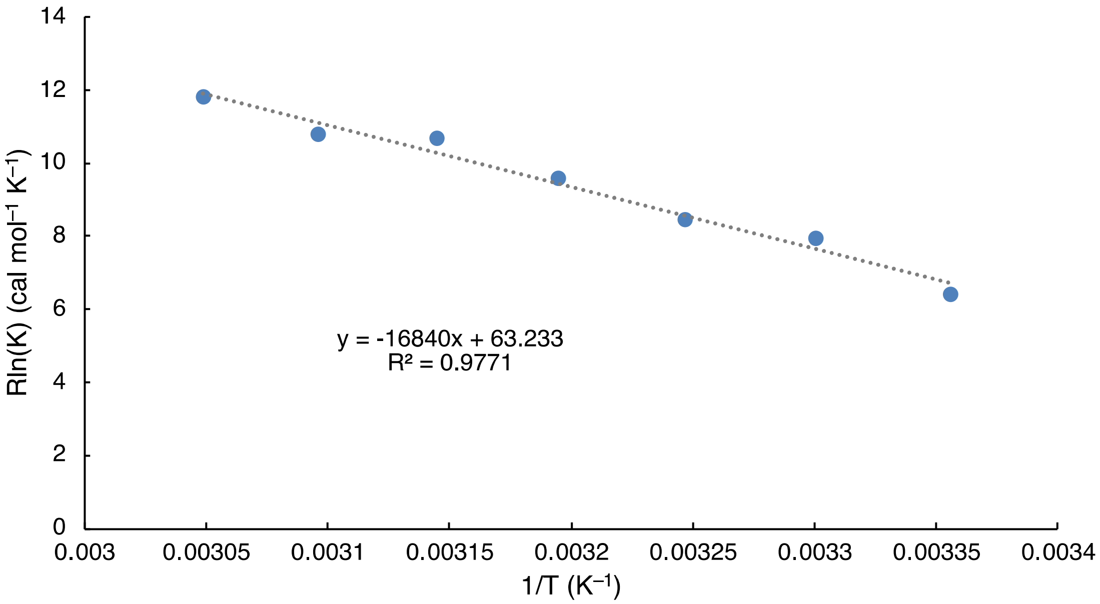

Figure S7: van't Hoff plot of $[Ga_4L_6]^{12-}$ and NEt_4^+ in D_2O.

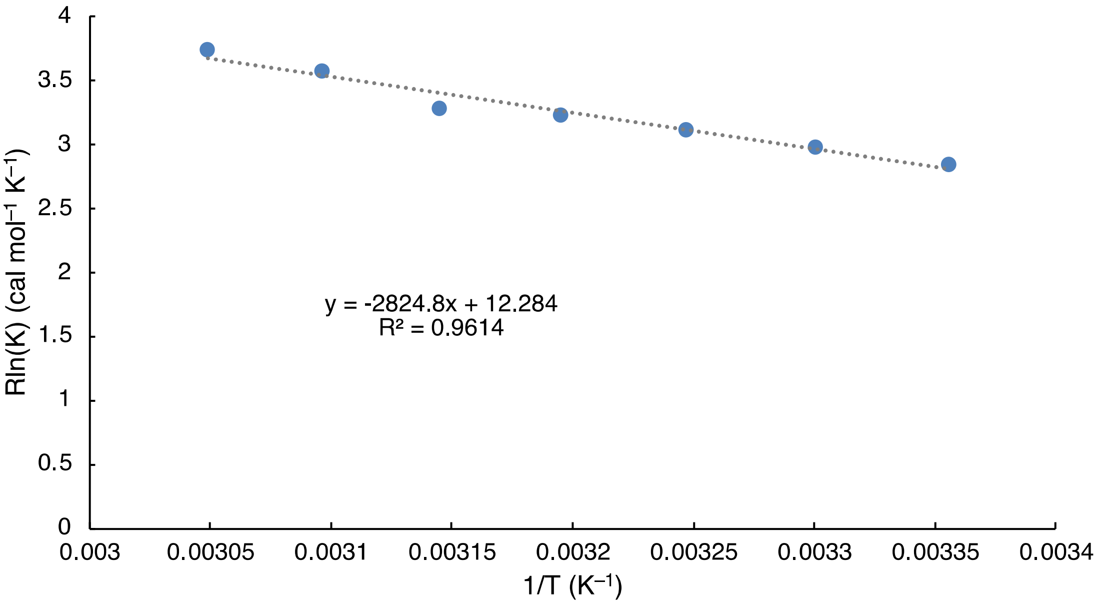

Figure S8: van't Hoff plot of $[In_4L_6]^{12-}$ and NEt_4^+ in DMSO-d_6.

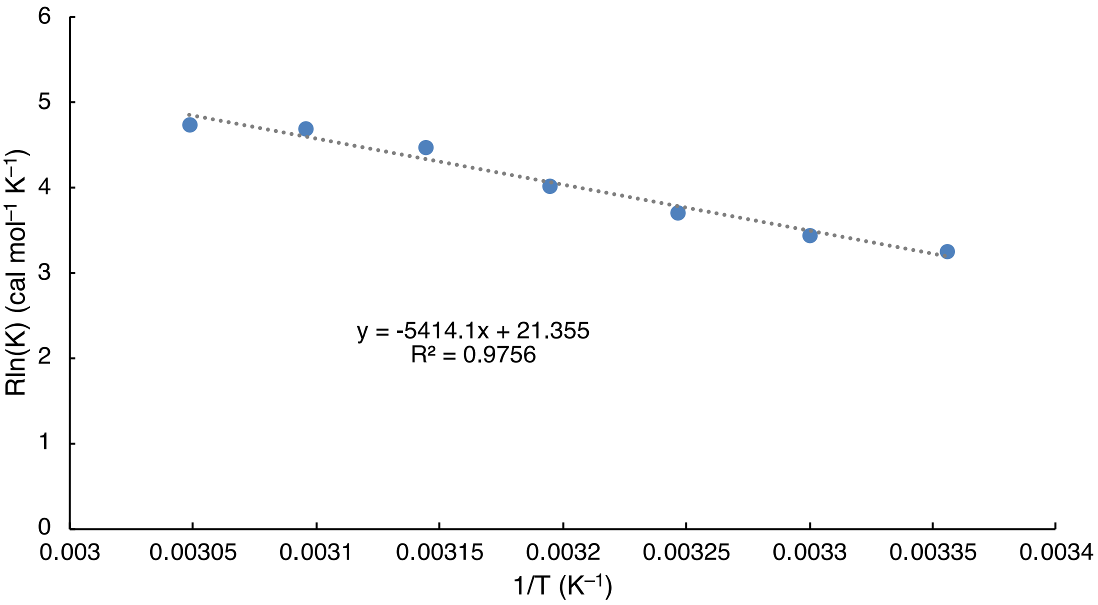

Figure S9: van't Hoff plot of $[In_4L_6]^{12-}$ and NEt_4^+ in 20% DMSO-d_6 in D_2O.

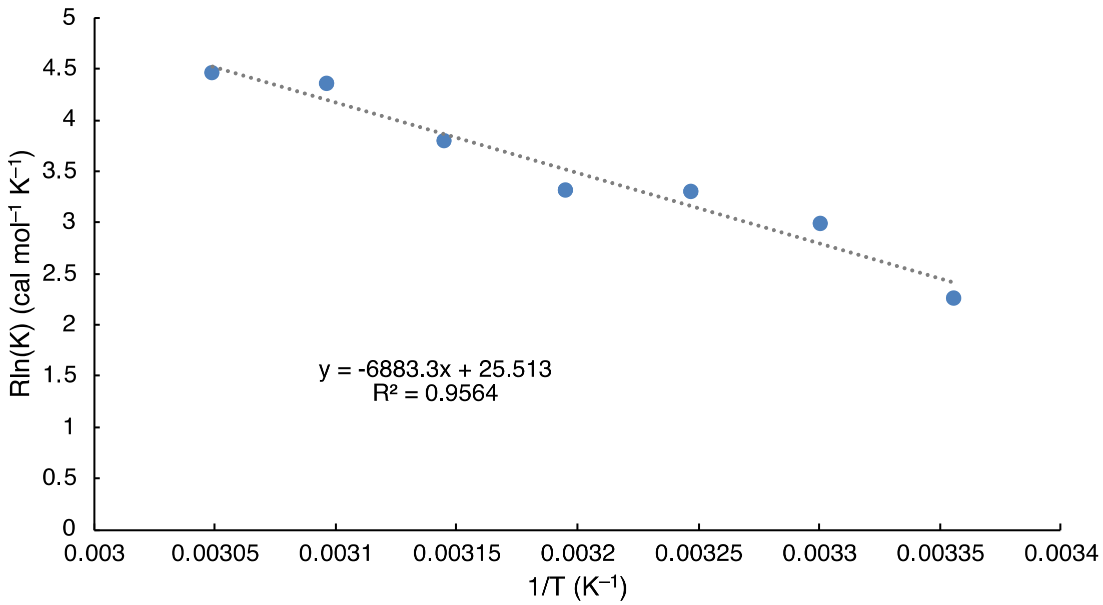

Figure S10: van't Hoff plot of $[In_4L_6]^{12-}$ and NEt_4^+ in 10% DMSO-d_6 in D_2O.

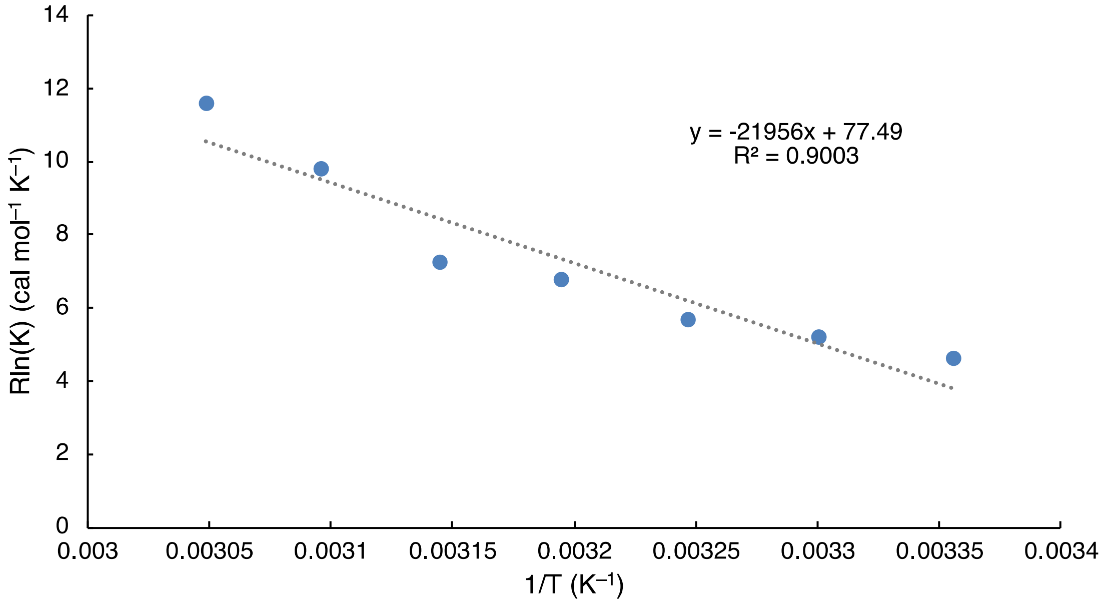

Figure S11: van't Hoff plot of $[In_4L_6]^{12-}$ and NEt_4^+ in D_2O.

Summary of van't Hoff data

Table S3. Enthalpy of $[Ga_4L_6]^{12-}$ (Ga) and $[In_4L_6]^{12-}$ (In) for different percentages of DMSO-d_6. Standard error for the last digit is reported in parentheses, computed from the duplicate experiments.

%DMSO	**ΔH** (Ga) [$kcal·mol^{-1}$]	**ΔH** (In) [$kcal·mol^{-1}$]
0	16.8(2)	22.0(4)
5	6.6(3)	
10	6.1(4)	7.1(4)
15	8.4(4)	
20	9.8(5)	5.4(5)
100	4.6(4)	2.8(5)

Table S4. Entropy of $[Ga_4L_6]^{12-}$ (Ga) and $[In_4L_6]^{12-}$ (In) for different percentages of DMSO-d_6. Standard error for the last digit is reported in parentheses, computed from the duplicate experiments.

%DMSO	**ΔS** (Ga) [$cal·mol^{-1}·K^{-1}$]	**ΔS** (In) [$cal·mol^{-1}·K^{-1}$]
0	63(3)	76(5)
5	24(3)	
10	23(4)	24(4)
15	32(5)	
20	36(5)	20(4)
100	15(3)	12(3)

Competitive guest binding experiment

Further experiments were conducted to determine the relative binding affinity of NEt_4^+ to the two hosts.

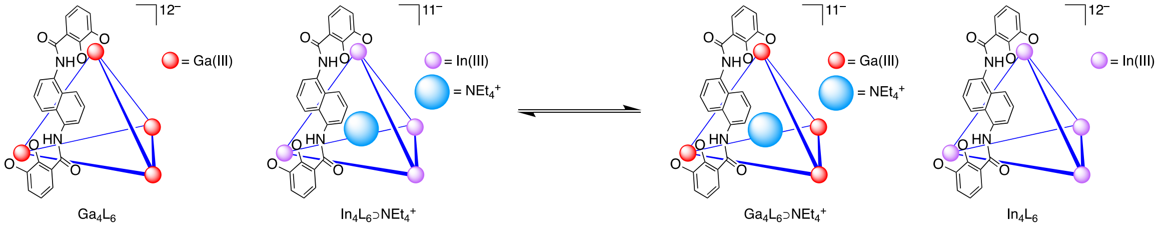

Measurements were performed with 2 mM Ga_4L_6, 2 mM In_4L_6, 2 mM NEt_4Cl:

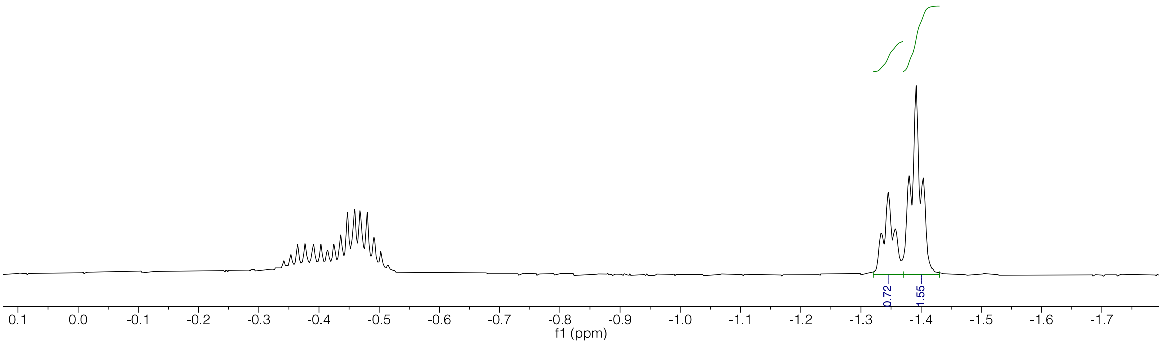

Fig. S12. Example of an NMR spectrum for competitive guest binding experiment. NMR detail of the encapsulated guest peaks.

$NEt_4^+ \subset In_4L_6$ peaks are shifted further upfield than $NEt_4^+ \subset Ga_4L_6$.

Below, data is shown for van't Hoff experiments of the competitive guest binding, in pure water and in a mixture of water and DMSO. **Table S5** and **Table S7** show the ratio of the guest bound in each host at different temperatures.

Table S5. Competitive guest binding experiment of $[Ga_4L_6]^{11-}$and $[In_4L_6]^{11-}$ in 100% D_2O at temperatures T.

**T** [K]	**$[NEt_4^+ \subset In_4L_6]$: $[NEt_4^+ \subset Ga_4L_6]$**
298	1.58
303	1.64
308	1.73
313	1.91
318	1.96
323	2.03

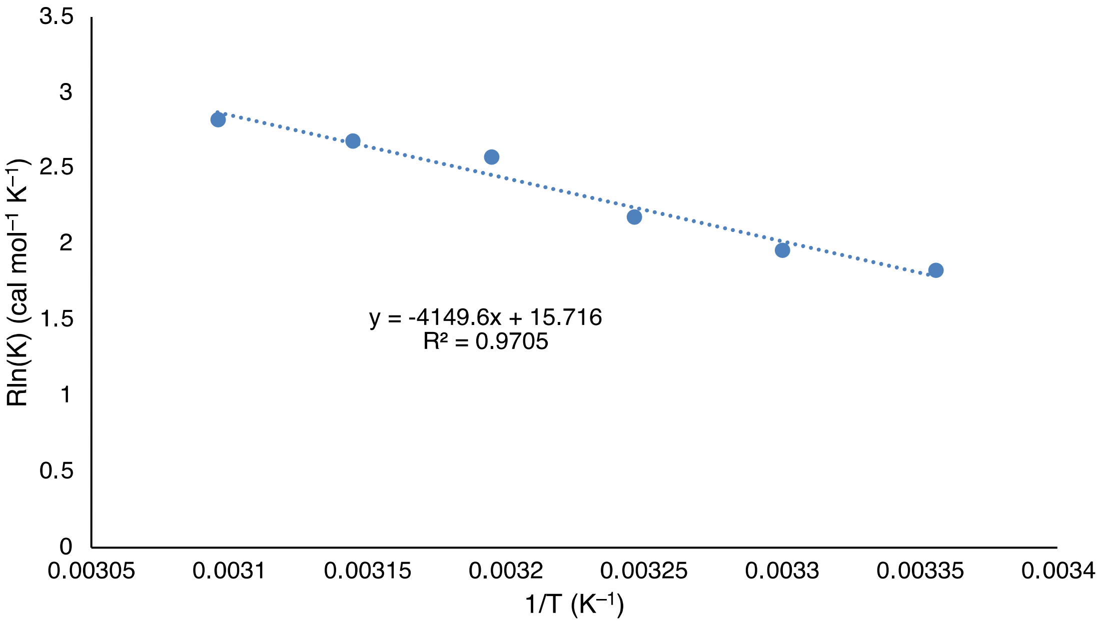

Fig. S13. Van't Hoff plot in 100% D_2O.

Table S6. Equilibrium constants K_{eq} of competitive guest binding experiment at temperatures T in 100% D_2O.

**T** [K]	**K_{eq}** [no unit]
298	2.51
303	2.68
308	3.00
313	3.66
318	3.85
323	4.14

Table S7. Competitive guest binding experiment of $[Ga_4L_6]^{11-}$and $[In_4L_6]^{11-}$ in 20% DMSO-d_6 and 80% D_2O at different temperatures T.

**T** [K]	**[$NEt_4^+ \subset In_4L_6$] : [$NEt_4^+ \subset Ga_4L_6$]**
298	1.42
303	1.67
308	1.84
313	1.89
318	2.07
323	2.12

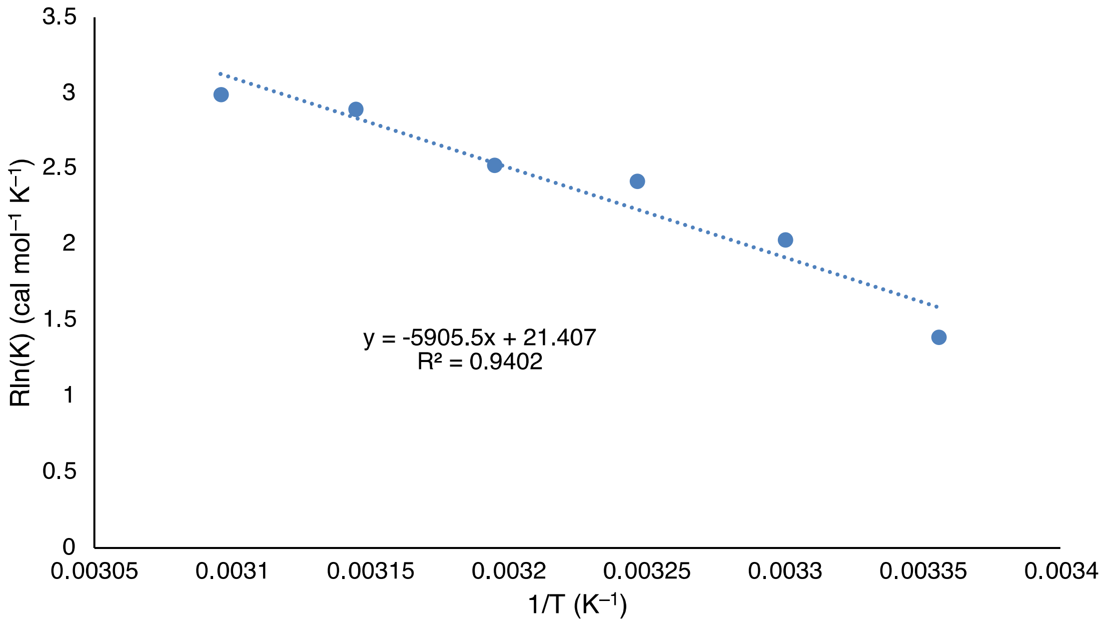

Fig. S14: Van't Hoff plot in 20% DMSO-d_6 and 80% D_2O.

Table S8: Equilibrium constants K_{eq} of competitive guest binding experiment at temperatures T in 20% DMSO-d_6 and 80% D_2O.

**T** [K]	$\mathbf{K_{eq}}$ [no unit]
298	2.02
303	2.78
308	3.37
313	3.56
318	4.28
323	4.50

THz-FIR Spectroscopy

Method and Materials for THz-FIR Spectroscopy Experiments

All samples of the supramolecular hosts $[Ga_4L_6]^{12-}$ (Ga-host) and $[In_4L_6]^{12-}$ (In-host) were prepared in a nitrogen-purged glovebox with and without the guest molecule tetraethylammonium chloride (NEt_4Cl) and dissolved in different aqueous solutions right before each measurement:

First, the Ga-host solved in degassed ultrapure water with and without the encapsulated guest was prepared with a molar concentration of c ≈ 20 mM, measured and analyzed as reported by Sebastiani *et al* (3). Then, Ga-host and In-host (c ≈ 10 mM) were dissolved in an aqueous, degassed 10% DMSO solution (DMSO:H_2O, 1:9, V/V) with and without NEt_4Cl, measured, analyzed and compared to the Ga-host spectrum. All THz-FIR absorption measurements of the samples were probed in a wavenumber range of 30 – 50 cm^{-1} (1.5 – 12 THz) as an average over 128 scans with a resolution of 2 cm^{-1}, using a Bruker Vertex 80V FTIR spectrometer. Fig. S15 shows a schematic setup:

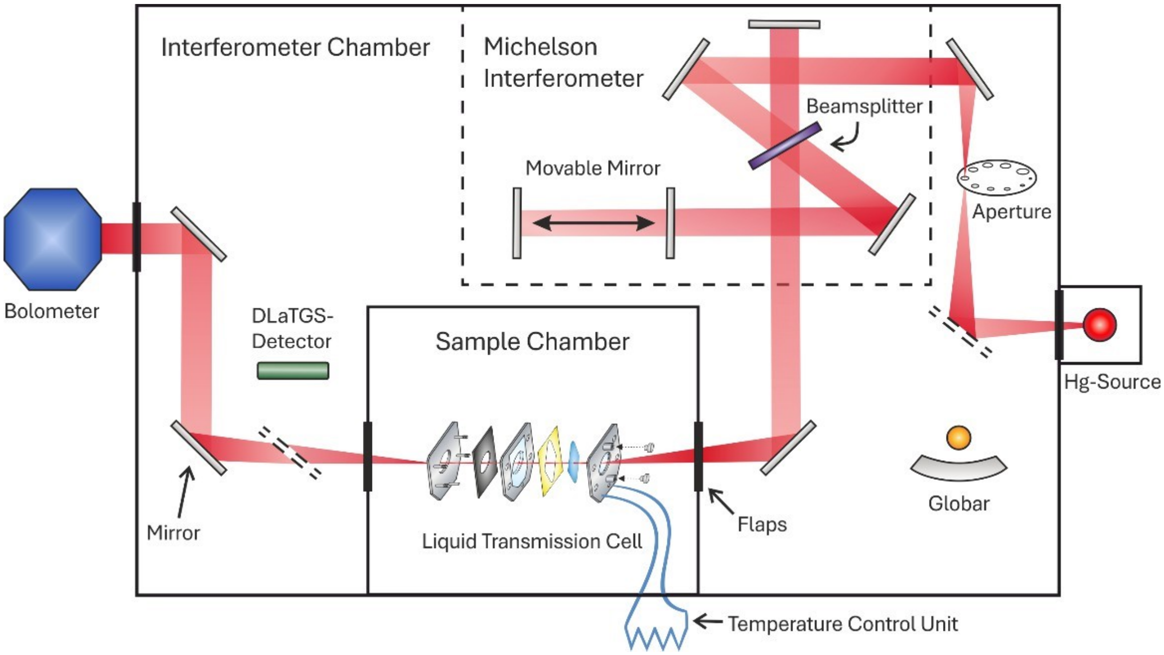

Fig. S15. Schematic setup of the FTIR spectrometer with a liquid transmission cell. An adjustable aperture guides the THz radiation from the Hg source to the Michelson interferometer beamsplitter, where the two interfering beams are passed through the sample cell to the silicon bolometer. The detector has a detection range of 30 – 680 cm^{-1}.

A mercury arc lamp was used as a light source for the THz-range and the beam was limited by an aperture with a diameter of 4.0 mm. The radiation was guided into the sample chamber through a Michelson Interferometer with a Mylar Multilayer beamsplitter. Sample chamber and interferometer are divided into separate compartments that can be purged and evacuated individually or simultaneously at around 10 hPa. The chambers are separated by radiation-transmissive flaps made of high-density polyethylene (HDPE), which are only permeable to THz-radiation. During the measurement process, the sample chamber is purged with nitrogen to minimize interfering signals

caused by remaining humidity in the sample beam path and the degradation of the samples. The beam was passed through a temperature-controlled Bruker liquid transmission cell (Diamond Materials, GmbH, Fig. S7) and collected by a silicon bolometer that was previously cooled by liquid helium.

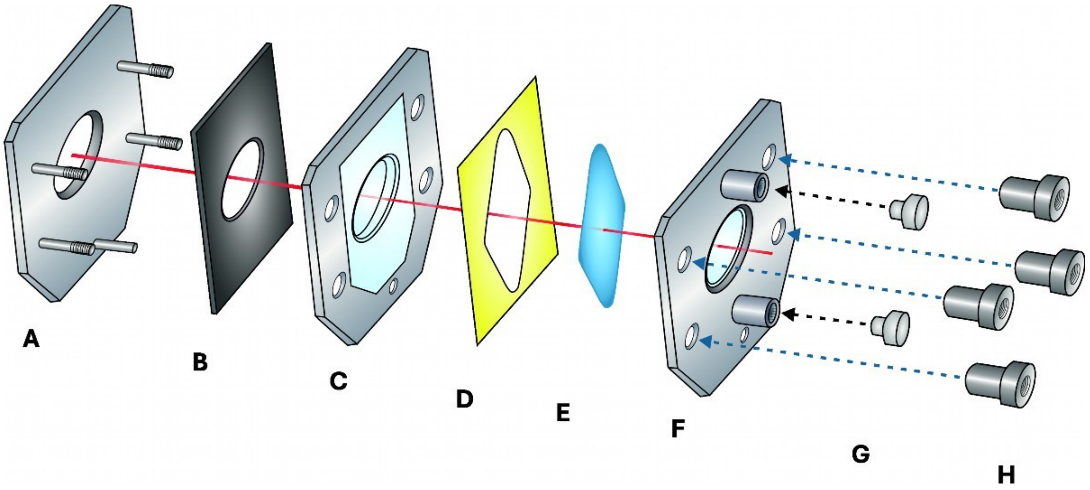

Fig. S16. Schematic setup of the Bruker liquid transmission cell (Diamond Materials, GmbH). A: Back plate. B: Neoprene gasket. C: Chemical vapor deposition-grown (CVD-) diamond window. D: Kapton spacer. E: Liquid sample. F: Front plate with CVD-diamond window and sample inlet openings. G: Caps. H: Screw caps.

The sample layer thickness was regulated by a fixed spacer size (Fig. S16, D) with a nominal thickness of d ~25 μm. The data was recorded at a constant temperature T of 293 ± 0.2 K (temperature control unit: Ministrat 240, Huber Kältemaschine AG) and a humidity level below 5%. By means of Fourier transformation, the measured interferograms were lastly converted into frequency-dependent absorption spectra by use of the software OPUS (Bruker). The densities of all samples were measured directly after the spectroscopic experiments at 20 °C with a DMA 58 density meter (Anton Paar).

Models of DMSO:H_2O and Bulk Water Absorption Coefficients

To obtain the absolute absorption coefficient $\alpha_{10\%\,DMSO}(\upsilon)$ of the aqueous DMSO-solution and $\alpha_{DMSO}(\upsilon)$ of the pure DMSO the samples were first probed with two different spacers of the nominal layer thicknesses d_1 ~13 µm and d_2 ~25 µm: We deduced $\alpha_{DMSO}(\upsilon)$ by:

$$\alpha_{DMSO}(\nu) = \frac{1}{(d_2 - d_1)} \cdot ln\left(\frac{I_{DMSO,1}(\nu)}{I_{DMSO,2}(\nu)}\right) \quad (S2)$$

The obtained Spectrum of $\alpha_{10\%\,DMSO}(\upsilon)$ was smoothened by using first-order interpolation. Both spectra were plotted in Fig. S8 (black, red).

A bulk water model was used to estimate the absolute absorption coefficient $\alpha_{H_2O}(\upsilon)$ of bulk water at 20 °C (Fig. S8, gray) and the number of water molecules in the cavities of the supramolecular hosts. A series of temperature-dependent THz absorption measurements was performed in our laboratory and related to the water data of Bertie and Lan.(4) One Debye term and three damped harmonic oscillator terms were used to fit the extinction coefficient of bulk water $\varepsilon_{H_2O}(\upsilon)$ at 20 °C, according to:

$$\varepsilon_{H_2O}(\nu) = \left(\frac{\alpha_{H_2O}(\nu)}{c^0_{H_2O}}\right) = \varepsilon_{LF}(\nu) + \varepsilon_{MID}(\nu) + \varepsilon_{HF}(\nu) \quad (S3)$$

$$\varepsilon_{LF}(\nu) = \frac{\tilde{a}_0 e^{-\frac{\nu}{\tilde{\nu}_{CO,i}}}}{\pi\left(\nu^2 + \frac{\tilde{w}_0(0)^2}{\pi^2}\right)} \quad (S4)$$

$$\varepsilon_{MID}(\nu) + \varepsilon_{HF}(\nu) = \sum_{i=1}^{3} \frac{\tilde{a}_i w_i^2(\nu)}{4\pi^3\left(\left(\tilde{\nu}_{b,i}^2 + \frac{w_i^2(\nu)}{4\pi^2} - \nu^2\right) + \frac{\nu^2 w_i^2(\nu)}{\pi^2}\right)}\nu^2 \quad (S5)$$

with

$$\tilde{w}_i(\tilde{\nu}) = \tilde{w}_i(0)e^{-\frac{\nu}{\tilde{\nu}_{co,i}}} \quad (S6)$$

The parameters of bulk water are given in Ref.(4), while $\tilde{w}_i$ describes a frequency dependent damping factor. The total error of the model in Eqs.S3 – S5 is below 5%.

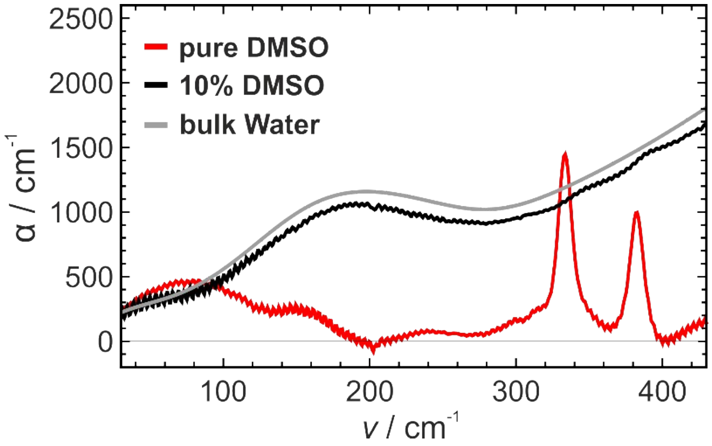

Fig. S17. Absorption spectra $\alpha_{10\%\ DMSO}(\upsilon)$ of pure DMSO (red), the aqueous solution 10% DMSO (DMSO:H2O, 1:9, V/V, black) and $\alpha_{H_2O}(\upsilon)$ of bulk water (gray). Spectra are displayed from 30 cm^{-1} to 430 cm^{-1}.

Both bulk water (gray) and 10% DMSO (black) show the intermolecular H-bond stretching mode around 190 cm^{-1}, followed by an increase in the absorption spectrum with higher frequencies, which are the onset of the librational mode centered at 600 – 700 cm^{-1}.(5) In the 10% DMSO spectra (black) despite of the small amount of DMSO in the solution, two very weak modes can be observed in the range from 330 cm^{-1} to 420 cm^{-1}. For comparison, we show the pure DMSO (red) spectrum with two intermolecular modes at 350 cm^{-1} and 390 cm^{-1}, while at lower frequencies a broader mode between 30 cm^{-1} and 200 cm^{-1} has been detected.

Analysis of THz-FIR Spectroscopy Experiments of Ga-host in ultrapure water

The absorption coefficient $\alpha_{sample}(\nu)$ (Eq. S7) was deduced using Lambert-Beer's Law. The cell thickness d ~25 µm was estimated by analyzing the interference patterns of the empty cell. I is transmitted intensities of the measured samples I_{sample}, and I_{H_2O} is the transmitted intensity of the reference bulk water at temperature T_0.

$$\alpha_{sample}(\nu) = -\frac{1}{d} \cdot ln\left(\frac{I_{sample}(\nu)}{I_{H_2O}(\nu)}\right) + \alpha_{H_2O}(\nu) \quad \text{(S7)}$$

We measured the solvated $[Ga_4L_6]^{12-}$ and compared the results to the encapsulated NEt_4^+, both at 20 °C ± 0,2°C. The equation S7 was used to reduce artefacts due to reflection of the cell windows. The absorption coefficient of bulk water $\alpha_{H_2O}(\nu)$ was obtained by fitting a water spectrum at a specific temperature T. Additionally, the spectra were corrected by a scaled water vapor spectrum, to minimize the absorption of residual air in the beam path.

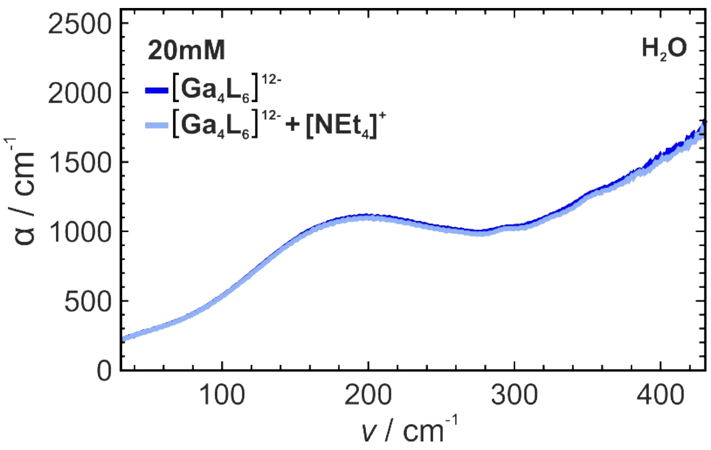

Fig. S18. Absorption spectra $\alpha_{sample}(\nu)$ of $[Ga_4L_6]^{12-}$ (20 mM) filled with water (blue) or guest cation NEt_4^+ (light blue) in ultrapure water.

The effective difference absorption coefficient of the solute and its hydration water $\Delta\alpha^{Eff}(\nu)$ was obtained, as described in Eq. S8:

$$\Delta\alpha^{Eff}(\nu) = \alpha_{sample}(\nu) - \left(\frac{c_{H_2O}}{c^0_{H_2O}}\right) \cdot \alpha_{H_2O}(\nu) \quad \text{(S8)}$$

Here, c_{H_2O} represents the molar concentrations of water in the solute and water mixture, while $c^0_{H_2O}$ describes the molar concentration of the ultrapure water, based on mass density measurements at 20 °C. This ensured concentration-dependent changes in the apparent molar

volume of the solute were taken into account.(6) The obtained $\Delta\alpha^{Eff}(\upsilon)$ spectra of the Ga-host with and without NEt_4Cl, dissolved in ultrapure water (c ≈ 20 mM) is shown in Figure 2 (bottom: blue and light blue) in the main text.

Analysis of THz-FIR Spectroscopy Experiments of Ga- and In-host in 10% DMSO

The absorption coefficient spectra of $[Ga_4L_6]^{12-}$ and $[In_4L_6]^{12-}$ with and without the encapsulated guest in 10% DMSO solution, were obtained in a similar way as for the Ga-host in ultrapure water (see above, Eq. S7). As reference we used a 10% DMSO solution instead of bulk water:

$$\alpha_{sample}(\nu) = -\frac{1}{d} \cdot ln\left(\frac{I_{sample}(\nu)}{I_{10\%\ DMSO}(\nu)}\right) + \alpha_{10\%\ DMSO}(\nu) \quad \text{(S9)}$$

Here, $I_{10\%\ DMSO}$ describes the transmitted intensities for the reference DMSO:H_2O (1:9, V/V) at a fixed temperature T, while $\alpha_{10\%\ DMSO}(\nu)$ represents the absolute absorption coefficient spectrum of 10% DMSO that was measured and fitted by first-order interpolation (see Eq.S2, Fig. S17 (black)).

In Fig. S21 and Fig. S22 the obtained absorption coefficient spectra for both Ga- and In-host with and without NEt_4Cl in 10% DMSO are shown:

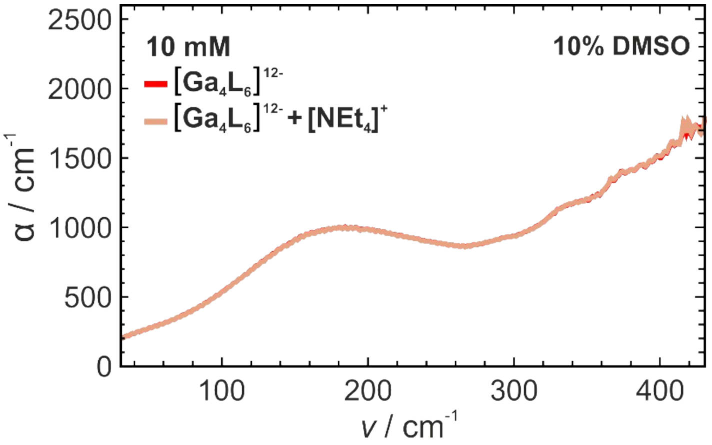

Fig. S19. Absorption coefficient spectra $\alpha_{sample}(\nu)$ of $[Ga_4L_6]^{12-}$ (10 mM) filled with aqueous DMSO solution (red) or encapsulated guest (light red).

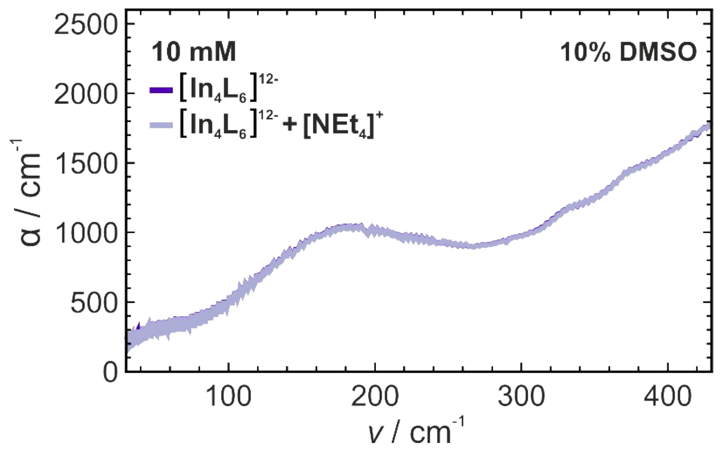

Fig. S20. Absorption coefficient spectra $\alpha_{sample}(v)$ of $[In_4L_6]^{12-}$ (10 mM) filled with aqueous DMSO solution (purple) or encapsulated guest (light purple).

Datasets for THz-FIR Spectroscopy Experiments of Ga- and In-host:

To ensure sample stability and reproducibility of the data shown, the measurements were recorded three times for each host-guest system with and without guest in 10% DMSO solution. The spectra obtained are shown below for comparison:

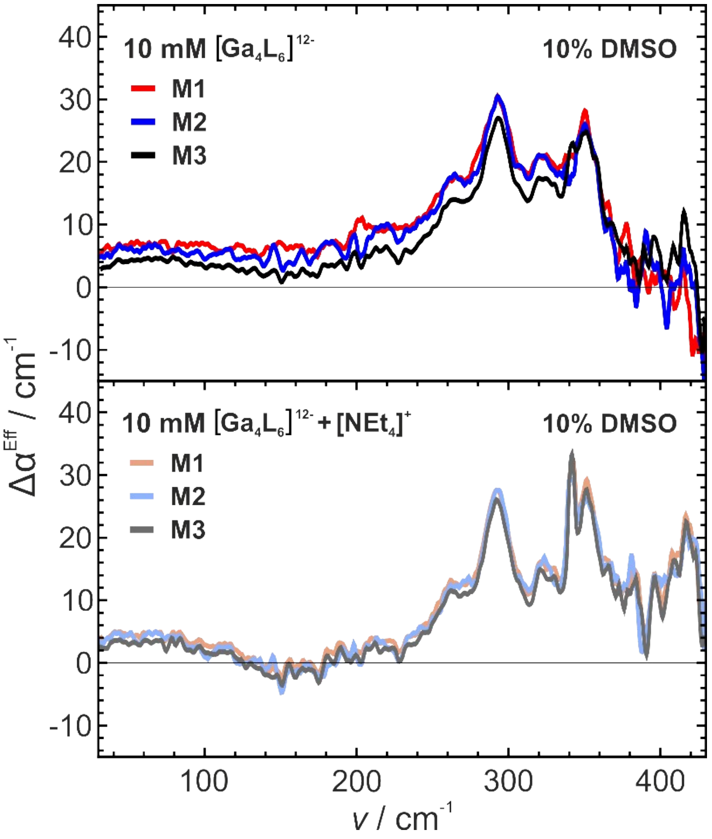

Fig. S21. Comparison of independent measurements (M1-3) for the effective difference absorption spectra $\Delta\alpha^{Eff}(\upsilon)$ of $[Ga_4L_6]^{12-}$ (10 mM) dissolved in 10% DMSO without guest (red, blue, black) or with encapsulated guest cation NEt_4^+ (light red, light blue, light gray).

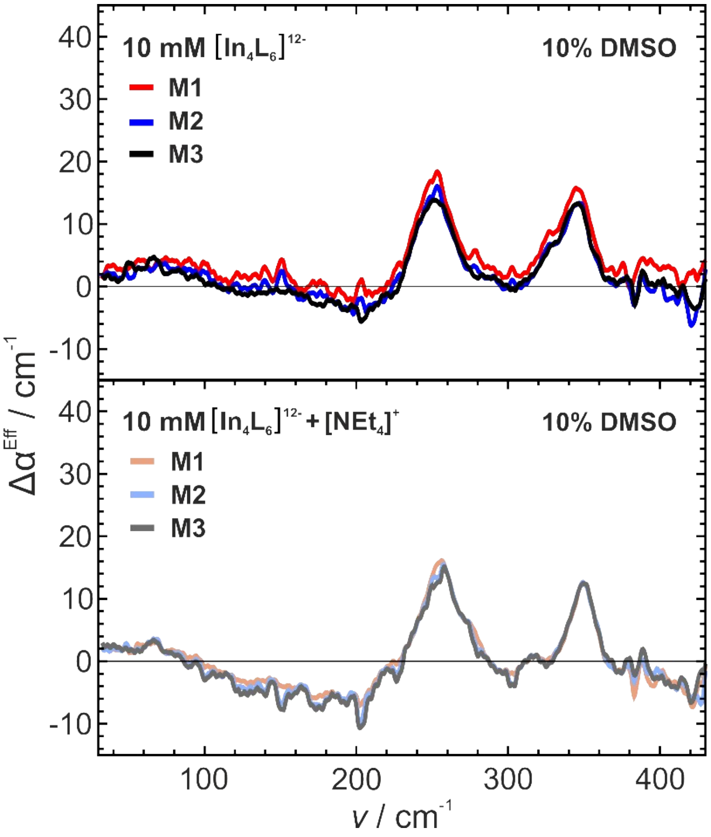

Fig. S22. Comparison of independent measurements (M1-3) for the effective difference absorption spectra $\Delta\alpha^{Eff}(\upsilon)$ of $[In_4L_6]^{12-}$ (10 mM) dissolved in 10% DMSO without guest (red, blue, black) or with encapsulated guest cation NEt_4^+ (light red, light blue, light gray).

Both data sets for $[Ga_4L_6]^{12-}$ and $[In_4L_6]^{12-}$ dissolved in 10 % DMSO solution show similar intensities and behaviors. The comparison shows that the data is reproducible.

In a similar way two measurements each for $[Ga_4L_6]^{12-}$ (20 mM) dissolved in ultrapure water without guest (Fig. S14,) and with guest (Fig. S14,) were measured at two days:

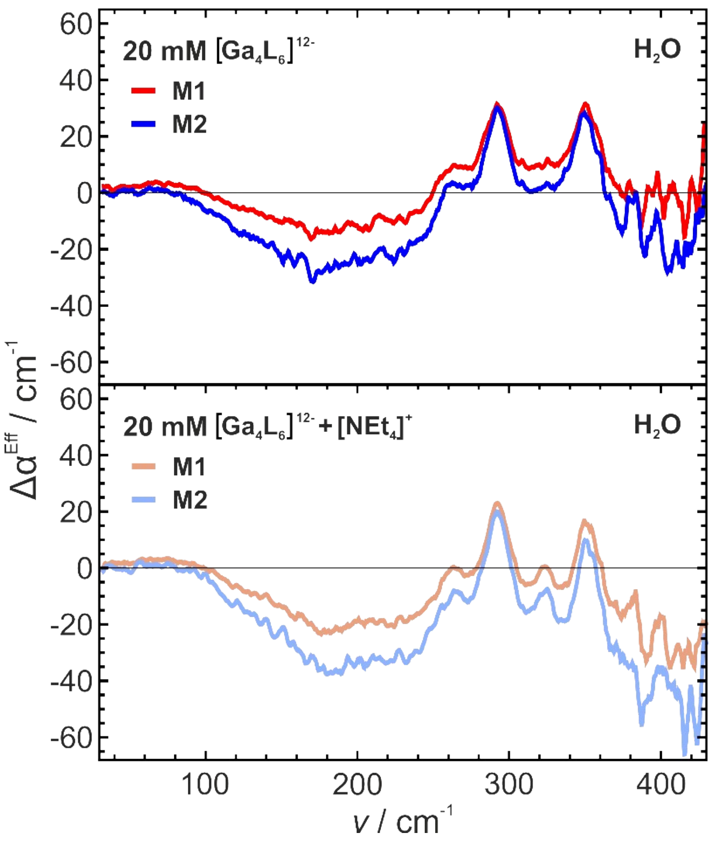

Fig. S23. Comparison of two consecutive measurements (M1-2). Plotted is $\Delta\alpha^{Eff}(v)$ of $[Ga_4L_6]^{12-}$ (20 mM) dissolved in ultrapure water without guest (red and blue) or with encapsulated guest cation NEt_4^+ (light red and light blue).

We found no significant deviations. The slight offset can be explained by the fact that the background was recorded at different days. To account for this offset, the host-guest systems in a 10% DMSO solution were measured on the same day, which minimized the systematic error.

SI References

1. G. R. Fulmer, *et al.*, NMR chemical shifts of trace impurities: Common laboratory solvents, organics, and gases in deuterated solvents relevant to the organometallic chemist. *Organometallics* **29**, 2176–2179 (2010).
2. Q. N. N. Nguyen, *et al.*, Source of Rate Acceleration for Carbocation Cyclization in Biomimetic Supramolecular Cages. *J Am Chem Soc* **144**, 11413–11424 (2022).
3. F. Sebastiani, *et al.*, An isolated water droplet in the aqueous solution of a supramolecular tetrahedral cage. *Proc Natl Acad Sci U S A* **117**, 32954–32961 (2020).
4. J. E. Bertie, Z. Lan, Infrared intensities of liquids XX: The intensity of the OH stretching band of liquid water revisited, and the best current values of the optical constants of $H_2O(1)$ at 25 °C between 15,000 and 1 cm^{-1}. *Appl Spectrosc* **50**, 1047–1057 (1996).
5. V. C. Nibali, M. Havenith, New insights into the role of water in biological function: Studying solvated biomolecules using terahertz absorption spectroscopy in conjunction with molecular dynamics simulations. *J Am Chem Soc* **136**, 12800–12807 (2014).
6. G. Schwaab, F. Sebastiani, M. Havenith, Ion Hydration and Ion Pairing as Probed by THz Spectroscopy. *Angewandte Chemie International Edition* **58**, 3000–3013 (2019).
